# Enriched knowledge representation in biological fields: a case study of literature-based discovery in Alzheimer’s disease

**DOI:** 10.1186/s13326-025-00328-3

**Published:** 2025-03-20

**Authors:** Yiyuan Pu, Daniel Beck, Karin Verspoor

**Affiliations:** 1 School of Computing and Information Systems, The University of Melbourne, 700 Swanston St, Melbourne, VIC 3053 Victoria Australia; 2 School of Computing Technologies, RMIT University, 124 La Trobe St, Melbourne, VIC 3000 Victoria Australia

**Keywords:** Knowledge representation, Literature-based Discovery, Knowledge graph, Swanson’s ABC model, Link prediction, Alzheimer’s Disease, Pairwise relationship, Hypergraph, Nested relationship

## Abstract

**Background:**

In Literature-based Discovery (LBD), Swanson’s original ABC model brought together isolated public knowledge statements and assembled them to infer putative hypotheses via logical connections. Modern LBD studies that scale up this approach through automation typically rely on a simple entity-based knowledge graph with co-occurrences and/or semantic triples as basic building blocks. However, our analysis of a knowledge graph constructed for a recent LBD system reveals limitations arising from such pairwise representations, which further negatively impact knowledge inference. Using LBD as the context and motivation in this work, we explore limitations of using pairwise relationships only as knowledge representation in knowledge graphs, and we identify impacts of these limitations on knowledge inference. We argue that enhanced knowledge representation is beneficial for biological knowledge representation in general, as well as for both the quality and the specificity of hypotheses proposed with LBD.

**Results:**

Based on a systematic analysis of one co-occurrence-based LBD system focusing on Alzheimer’s Disease, we identify 7 types of limitations arising from the exclusive use of pairwise relationships in a standard knowledge graph—including the need to capture more than two entities interacting together in a single event—and 3 types of negative impacts on knowledge inferred with the graph—Experimentally infeasible hypotheses, Literature-inconsistent hypotheses, and Oversimplified hypotheses explanations. We also present an indicative distribution of different types of relationships. Pairwise relationships are an essential component in representation frameworks for knowledge discovery. However, only 20% of discoveries are perfectly represented with pairwise relationships alone. 73% require a combination of pairwise relationships and nested relationships. The remaining 7% are represented with pairwise relationships, nested relationships, and hypergraphs.

**Conclusion:**

We argue that the standard entity pair-based knowledge graph, while essential for representing basic binary relations, results in important limitations for comprehensive biological knowledge representation and impacts downstream tasks such as proposing meaningful discoveries in LBD. These limitations can be mitigated by integrating more semantically complex knowledge representation strategies, including capturing collective interactions and allowing for nested entities. The use of more sophisticated knowledge representation will benefit biological fields with more expressive knowledge graphs. Downstream tasks, such as LBD, can benefit from richer representations as well, allowing for generation of implicit knowledge discoveries and explanations for disease diagnosis, treatment, and mechanism that are more biologically meaningful.

**Supplementary Information:**

The online version contains supplementary material available at 10.1186/s13326-025-00328-3.

## Background

Literature-based discovery (LBD) aims to identify knowledge that is implicit in findings and assertions in published literature [27]. Given the large and growing volumes of existing scientific literature, LBD has become increasingly important to help researchers propose novel, plausible and non-trivial scientific hypotheses by making connections between papers. Highlighting the need for tools to help navigate these connections, a search in PubMed with a keyword “Alzheimer’s Disease”^1^ returns 223,422 results at the time of this study.

In his pioneering LBD work, Swanson [30] revealed the hidden knowledge of Fish oil ameliorating Raynaud’s Syndrome by connecting unrelated papers in the literature. In this initial case, Swanson found that a collection of Raynaud’s Syndrome papers showed that a successful treatment depends on changes in blood parameters, including lower blood viscosity, platelet aggregability, and vascular reactivity. In parallel, a collection of fish oil literature suggested that dietary fish oil can produce chemicals that have these same effects on blood parameter changes. Fish oil might then be expected to ameliorate Raynaud’s Syndrome. However, these two literature collections are independent. Defining “A” as dietary fish oil, “C” as amelioration of Raynaud’s Syndrome, and ‘B” as reduction of blood viscosity, platelet aggregability, and vascular reactivity, we can model the fish oil literature as stating that “A causes B”, and the Raynaud’s Syndrome literature as indicating that “B causes C”. Then, a hypothesis of “A causes C” is inferred, suggesting that dietary fish oil may help Raynaud’s Syndrome. This knowledge discovery process is framed as Swanson’s *ABC model*. This is employed as the foundation of LBD: an A-to-C hypothesis is derived from public and disconnected knowledge of A-to-B and B-to-C relationships, through transitive closure.

An LBD system is expected to identify plausible and testable hypotheses that encourage scientists towards further exploration [27, 29]. It consists of a literature-derived knowledge base and methods for automatic prediction of new knowledge from that knowledge base. An essential requirement of an LBD system is representation of knowledge in a uniform format that a computer can process. Existing LBD systems [22, 24] use pairwise relations between two terms as a basic building block to represent knowledge, resulting in *knowledge graphs*. A knowledge graph (KG) is a graph-based data model that accumulates and conveys real-world knowledge [12]. In a KG, nodes represent entities of interest (such as “fish oil” or “Raynaud’s Syndrome”), and edges represent relations between entities (such as “ameliorate”). After a KG is constructed, putative new connections between nodes (or hypotheses) are proposed with link prediction [20]. There are two major categories of KG-based LBD systems: co-occurrence based models and semantic-based models [11]. These models differ in how they define a relation. Co-occurrence based models represent a relationship between two entity mentions based on whether they co-occur in a given text span. Semantic-based models utilize a more specific definition: if a text span contains two entities with an identified relation, then it extracts this relation as a *predicate* between these terms. These predicates are usually directed, while co-occurrences are undirected by design.

**Fig. 1:**
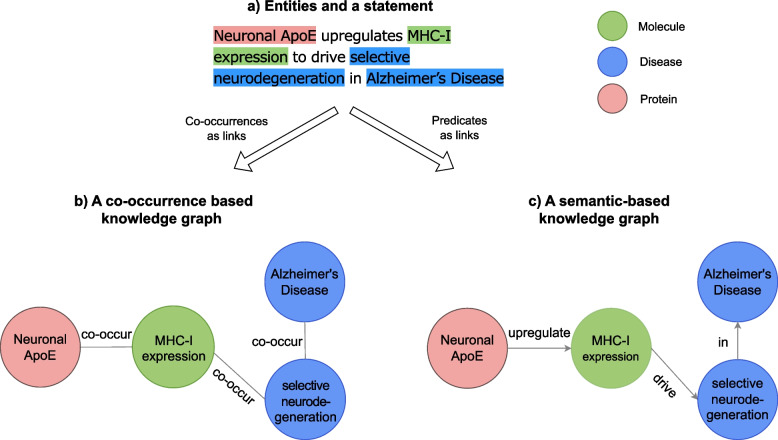
Example of knowledge graphs constructed from entities and relations in a statement. Top: terms and the corresponding knowledge discovery statement. Bottom left: a co-occurrence based knowledge graph. Bottom right: a semantic-based knowledge graph with predicates as links

Knowledge graphs with both co-occurrence based models [8, 13, 22] and semantic-based models [25, 38] have been explored in LBD. Nodes (or entities) can be terms extracted directly from literature text or a concept that is a unique identifier being mapped from a term. For instance, MeSH:D000544 is a unique identifier for a term Alzheimer’s Disease: both can be used as nodes in a knowledge graph. Edges (or links) can be either undirected, in the case of co-occurrences, or directed, when predicates are used.^2^ Figure 1 shows an example, where a discovery is present in the statement “Neuronal ApoE upregulates MHC-I expression to drive selective neurodegeneration in Alzheimer’s disease” (PMID 33958804). In this example, the terms are Neuronal ApoE, MHC-I expression, selective neurodegeneration, and Alzheimer’s disease. A co-occurrence based knowledge graph (Fig. 1b) represents the statement with three undirected links between four entities. A semantic-based knowledge graph (Fig. 1c) for the same statement consists of the same entities but connected through directed predicates.

With LBD as the context and motivation in this work, we argue that the standard knowledge graphs constructed for biological knowledge representation in general have substantial limitations. In Table 1, we show a representation for the discovery in Fig. 1 that better captures its interpretation by including a more abstracted relationship between a process involving several entities (Neuronal ApoE upregulating MHC-I expression) and the compound effect resulting from that process (selective neurodegeneration in Alzheimer’s Disease). This example highlights the limitations of pairwise relations in biological knowledge graphs. These limitations have important consequences for both the quality and the specificity of the discoveries that can be proposed with LBD. To the best of our knowledge, no previous study has explored incorporating richer representations into LBD. We aim to answer two research questions in this study:

- **RQ1** What types of limitations arise from the use of pairwise relations for biological knowledge representation?
- **RQ2** What is the impact of those limitations in LBD?

In addition to answering these two research questions, we also explore possible alternative strategies for knowledge representation that can mitigate these limitations for knowledge graphs and impacts for LBD.

We answer our research questions by systematically analyzing knowledge graphs built from a corpus in the context of an existing LBD system for Alzheimer’s Disease [22] (referred to as “AD-LBD system”). By examining these graphs, we illustrate which part of a discovery is not well captured with pairwise relations alone and categorize missing information into a set of limitation types. We also categorize different impact types of missing information in LBD. Finally, we suggest alternative knowledge representation strategies that can address those limitations and benefit downstream tasks for knowledge graphs such as LBD.

**Table 1.**
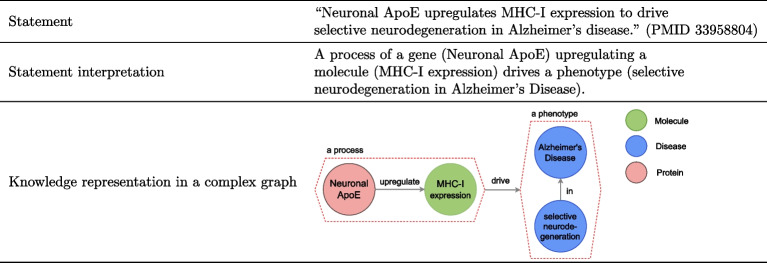
An example of a graph that is more biologically meaningful for the same statement in Fig. 1a. The graph more closely reflects the statement interpretation, compared to Fig. 1c

## Methods

This study aims at exploring the limitations of pairwise relationships in knowledge representation and investigate alternative mechanisms that can enhance knowledge graphs for the biological field. To achieve this, we present a case study in the domain of Alzheimer’s Disease (AD). First, we describe the corpus used in this study, which contains a collection of PubMed articles. We then briefly introduce an LBD system which was developed using this corpus and provide motivating examples for identifying the limitations of pairwise relationships. We finish this section by giving a theoretical description of the alternative knowledge representation mechanisms that we consider.

### Corpus

We consider a collection of over 16k papers in the domain of AD, published between 1977 and 2021 [22]. This collection was curated by a group of experts in the AD field^3^ and is centered around the amyloid hypothesis, which is a hypothesis that a build-up of the peptide amyloid beta ($A\beta$) in the brain causes AD. Articles were selected with keywords *Alzhiemer’s disease (AD), A-beta (or A*$\beta$)*, amyloid-beta (or amyloid-*$\beta$*), etc.* (See full keywords in Pu et al. [22]) The corpus was split into a training set, consisting of the papers from 1977 to 2020, and a test set, with papers published in 2021. We only consider the title and the abstract for each paper. Most of our analysis focuses on the test set, which contained a total of 56 papers; the training set was used only to train the LBD system in our prior work (described below).

### The AD-LBD system

AD-LBD [22] was an LBD system specifically designed to facilitate knowledge discovery in the context of Alzheimer’s Disease (AD). Like other LBD systems, it framed knowledge discovery as a link prediction task [31], where the goal was to infer new connections between concepts that were not previously linked in the knowledge graph. AD-LBD focused on AD-specific concepts and relationships, addressing a growing need for automated systems to navigate the expanding body of AD literature.

The system was based on a simple entity-based AD knowledge graph, where nodes were AD-specific concepts extracted automatically by an entity recognizer. Edges followed a co-occurrence model [11, 30]: if two concepts appeared in the same article, then a relation was established between two concepts. This co-occurrence based approach has been widely adopted in other LBD systems [24] for its simplicity and scalability. Inference of a new discovery was then done by predicting whether a new link exists between two concepts that have not yet had a connection.

The knowledge graph was initially built using the training set of the corpus described in “Corpus” section. Two automatic concept annotators were employed to extract entities from the articles, covering a wide range of AD-relevant domains:

- An AD-specific annotator called the NIO (Neuropsychological Integrative Ontology) annotator, covering the domains of neurodegenerative disease, brain areas, neuropsychological testing, and cognitive processes. This annotator was based on a dictionary-based approach validated in prior concept recognition work [10].
- A supplemental annotator called the PTC (PubTator Central) annotator, covering the domains of genes, genetic variants, disease, chemical, species, and cell line. This annotator utilizes existing annotations available in PubMed Central [35].

For the original AD-LBD system, evaluation was done by comparing predicted links with co-occurrence links present in the test set [22]. However, this study does not involve training and testing processes for link prediction models. Rather than analyzing our results quantitatively with training and testing datasets, we assess its conceptual link prediction capabilities by qualitatively probing its limitations in representing biological knowledge. For example, pairwise relationships in the knowledge graph were assessed for their capacity to support downstream tasks, such as hypothesis generation, by identifying missing links or oversimplified relationships that impede effective inference.

### Knowledge representation approaches

The AD-LBD system provided an initial approach to propose new hypotheses in the domain of Alzheimer’s Disease. While initial results were promising, a manual analysis showed substantial flaws, resulting from the simplifying assumption that discoveries can be modeled as pairwise relations (co-occurrence in that case). Here we describe two alternative, more powerful, approaches to knowledge representation that we argue are better suited to represent discoveries.

#### Hypergraphs

*Higher-order* interactions, or *n-ary* relations, are interactions among more than two elements. These provide important expressive power for knowledge representation. Pairwise interactions represented in a network have been argued as insufficient to capture collective actions that happen within a group of nodes for a complex system [1]. We therefore explore the value of higher-order relations for LBD.

Hypergraphs are a generalization of graphs that enable the description of higher-order interactions. A *hypergraph* is defined as a set of nodes and a set of *hyperedges*, which are subsets of related nodes [2]. A hyperedge allows for connecting more than two nodes, i.e., an arbitrary subset of entities, together into a higher-order relation. With a definition for hypergraph, a *simple graph* is a special hypergraph. Each hyperedge in a simple hypergraph is limited to having only two entities. In this study, we use hypergraphs as a more powerful alternative to a simple graph to represent interactions among entities.

To facilitate presentation of higher-order interactions, we adopt a reification-based data model for convenience. Specifically, each hyperedge is reified as a handle, effectively transforming it into a node. This approach enables the explicit representation of complex and multi-entity relationships as distinct entities in the hypergraph, allowing for richer semantic modeling and analysis. This approach has been suggested to support complex knowledge representation, including *graph annotations* (annotations of graphs) [18]. We remain agnostic, however, as to precisely how hypergraphs should be formalized in a specific implementation, noting that the semantics of reification has been questioned [3] and alternatives such as named graphs [6] may be more suitable.

**Fig. 2:**
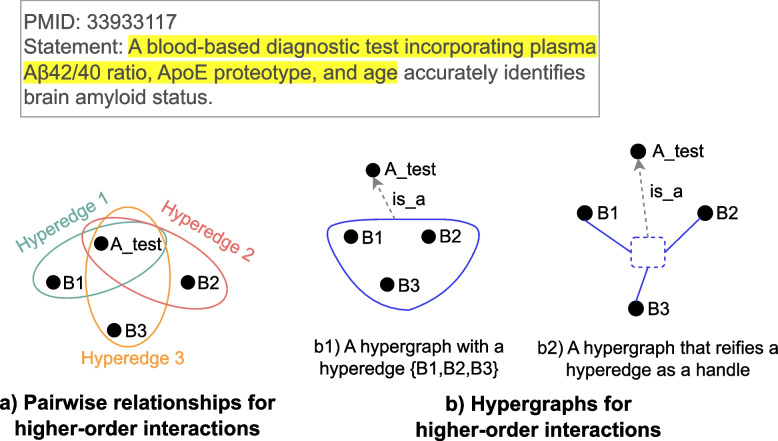
An example of using pairwise relationships and hypergraphs for higher-order interactions. A_test: A blood-based diagnostic test, B1: plasma A$\beta$42/40 ratio, B2: ApoE proteotype, B3: age. Upper: A statement in PMID 33933117 with a highlighted subject, which is to be represented with pairwise relationships and hypergraphs. Bottom left (**a**): Pairwise relationships that represent higher-order interactions as special hyperedges. Each hyperedge has only two entities: 1) Hyperedge 1: {A_test,B1}, 2) Hyperedge 2: {A_test,B2}, and 3) Hyperedge 3: {A_test,B3}. Bottom middle (b1): Hypergraphs that represent higher-order interactions as a hyperedge {B1,B2,B3}. The hyperedge is pointed to the node A_test with a relation of is_a. Bottom right (b2): A hyperedge in b1 is reified as a handle. The handle is pointed to the node A_test with a relation of is_a

A comparison between hypergraphs and pairwise relationships for higher-order interactions is shown for a specific example in Fig. 2. For a complex entity in the statement, a simple graph (Fig. 2a) only conveys pairwise relationships between each biomarker and a blood-based diagnostic test, while a hypergraph (Fig. 2b) conveys the semantic information that a combination of three biomarkers makes up a blood-based diagnostic test. A hypergraph is constructed by first having a hyperedge among three biomarkers (Fig. 2b1), and then reifying the hyperedge as a handle, which is further pointed to a general node a blood-based diagnostic test (Fig. 2b2).

#### Nested relationships

A subject or an object in a discovery statement can itself be a combination of entities and relations, i.e., a triple or subgraph: we call this phenomenon a *nested relationship*. A nested relationship is a hierarchical structure, capturing relations that exist *between* facts or statements. This is distinct from the higher-order relationships discussed above, where the elements connected by a hyperedge are assumed to be individual nodes.

**Fig. 3:**
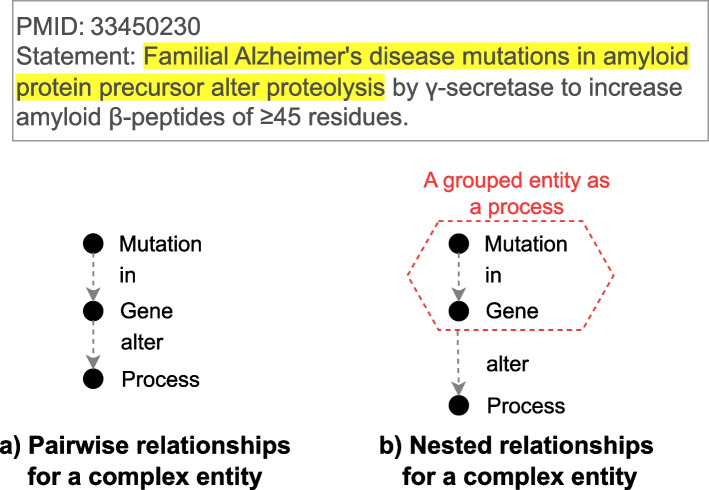
An example of using pairwise relationships and nested relationships for a complex entity. Mutation: Familial Alzheimer’s disease mutations, Gene: amyloid protein precursor, Process: proteolysis. Upper: A statement in PMID 33450230 with a highlighted complex entity. Bottom left (**a**): Represent a complex entity with pairwise relationships. The statement is represented with independent pieces of information: A mutation in a gene and a gene altering a process. Bottom right (**b**): Represent a complex entity with nested relationships. The statement is represented with dependent pieces of information. A process of a mutation in a gene alters another process

Our approach emphasizes the semantic representation of nested relationships in biological discovery statements. For example, in Fig. 3, the discovery statement describes how mutations in a gene alter a process. The subject Familial Alzheimer’s disease mutations in amyloid protein precursor in the statement is a complex entity that relates the mutations (Familial Alzheimer’s disease mutations) to the gene they occur in (amyloid protein precursor) through the trigger term “in”. In the pairwise representation (Fig. 3a), this complex structure is lost. The representation misses the critical relationship between the mutations and genes, only reflecting that a gene alters a process. However, nested relationships (Fig. 3b) group the mutations and the gene into a cohesive hierarchical entity, preserving their internal relationship. For the discovery statement, nested relationships show a process of mutations in a gene that alters another process, which is more precise than pairwise relationships. Another advantage of nested relationships is that they do not overwrite basic pairwise relationships. Instead, they extend the representational capacity of a knowledge graph by preserving pairwise relationships within the nested structure.

Unlike reified edges in a hypergraph structure (as described in “Hypergraphs” section) that treat a relationship as an entity, nested relationships maintain the hierarchical structure of entities and their dependent relationships. In Fig. 3b, a relationship between the mutation and the gene is not reified into an entity. Instead, these components are grouped to form a complex subject (mutations in a gene), which is directly linked to the process it alters. This approach preserves the integrity of the discovery statement without turning relationships into standalone entities. By representing hierarchical structures, nested relationships have three aspects of benefits: 1) capturing dependent relationships more accurately, 2) preserving contextual meaning within subjects or objects, and 3) providing more precise and semantically rich representations of discovery statements.

### Data exploration

We inspect and analyze the dataset of 56 articles (titles and abstracts) in the domain of Alzheimer’s Disease described in “Corpus” section. We manually probe the dataset to explore how best to respresent key findings and provide a systematic analysis of the limitations of pairwise relations. This study is exploratory and will inform the creation of formal and validated annotations over this corpus in future work.

First, we extract discovery statements from each paper: most of them contain only a single finding but some papers have more than one. Then, for each discovery, we extract “ideal” entities and relations by manually correcting concepts and relations from the AD-specific annotators in AD-LBD system (described in “The AD-LBD system” section). We represent these using either simple pairwise relationships or the more complex representations detailed in “Knowledge representation approaches” section where required.

We track our analysis in an Excel spreadsheet. We use the format “subject::predicate::object” for a pairwise relationship, and graph drawing software *draw.io*^4^ for graphs. The spreadsheet includes five columns, including a PMID, an article, natural language statements, extracted entities, and an alternative representation (corresponding to the “ideal representation” in the second step). Next, we create both a simple graph and a complex graph for each statement. A simple graph is illustrated with pairwise relationships only, while a complex graph is complemented with hypergraphs (“Hypergraphs” section) and nested relationships (“Nested relationships” section). The comparison of two graphs allows inspection of limitations of pairwise relationships, as well as impacts of these limitations on an LBD task. The analysis is publicly available on github^5^.

## Related work

### Biological information extraction

In biomedical information extraction, the objective is to use natural language processing techniques to transform information presented in the literature into structured data. These methods are often used in the context of LBD to transform literature into a knowledge graph. Entities and relations are core targets of these tools, mapping straightforwardly to a simple graph. However, it is also relatively common for biological information extraction tasks to target complex and nested relationships. This is framed as *event extraction* rather than *relation extraction*, since events may involve n-ary relations, or can be composed of relations among relations or events.

Examples of this can be found in the BioNLP 2009 [14] and BioNLP 2013 [21] shared tasks. For instance, in the BioNLP 2009 task, a key event type of interest is *Regulation*, where one biological process or event may regulate another. Consider the sentence “SQ 22536 suppressed gp41-induced IL-10 production in monocytes.” which, considering the two entities gp41 and IL-10 should be modelled via three statements shown in Table 2. This work provides evidence of the need for more complex representation of biological information to support discovery.

**Table 2:** Example of nested events from the BioNLP 2009 biomedical information extraction task [14], taken from [34]

ID	Type	Trigger	Theme	Cause
Event1	Negative_Regulation	suppressed	Event2	–
Event2	Positive_Regulation	induced	Event3	gp41
Event3	Gene_expression	production	IL-10	–

### Biological expression language

**Fig. 4:**
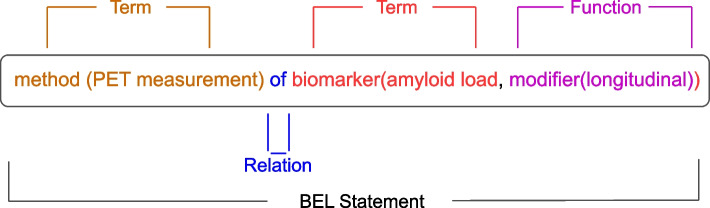
An example of a (simplified) BEL statement extracted from a complex entity PET measurement longitudinal amyloid load. The BEL statement consists of one method “PET measurement”, one biomarker “amyloid load”, one modifier for the biomarker “longitudinal”, and a relationship “of”

In addition to graphs, other knowledge representation approaches such as Biological Expression Language (BEL) statements can also be used to represent the semantics of texts on scale with a structure. BEL is a systems biology modeling language used to structure scientific findings in life sciences in a computable format. Scientific findings are described in BEL statements with BEL terms and relations in between [26]. Each BEL term is formed with biomedical entities and/or biological processes and functions that modify entities. A simplified BEL example in Fig. 4 illustrates the same nested relationship PET measurement of longitudinal amyloid load in a graph format in Fig. 8. For example, the BEL Term “method (PET measurement)” includes a biomedical entity “PET measurement” and a function “method()” that modifies the entity. A function can be also written as “modifier (longitudinal)” after an entity “amyloid load”, meaning that “amyloid load” is modified by “longitudinal”.

Like nodes in a knowledge graph being normalized to identifiers, BEL terms can also be normalized, which is expressed in namespace and associated identifiers. For instance, a protein “Heat Shock Transcription Factor 1” can be normalized to a namespace “HGNC” (HUGO Gene Nomenclature Committee) as “HSF1”. The normalized entity is represented as “p(HGNC:HSF1)” (p() for protein”).

### Hypergraphs in biological knowledge representation

Hypergraphs have been explored in biological knowledge representation. For instance, Feng et al. [9] adopted a hypergraph to capture multi-way relationships among genes and viral responses. In their hypergraph, significantly perturbed genes were hyperedges while biological samples with specific experimental conditions were nodes. Hypergraph measures were found to be superior to simple graph measures when identifying genes important to viral response. Murgas et al. [19] employed a hypergraph to represent diverse protein interactions within cells. Compared with a standard graph that only considers pairwise relationships for protein-protein interaction networks, their hypergraph model represented interactions among more than two entities, such as molecular pathways and dynamic cellular processes.

### Nested relations in knowledge graph reasoning

Recent advancements in knowledge graph reasoning have expanded to include nested relationships. For instance, Xiong et al. [36] introduced a NestE framework that handles nested relational structures in knowledge graphs. However, NestE is limited to representing triples with three nested sub-entities per nested entity, which may restrict its applicability to more complex hierarchical structures in biological knowledge graphs.

## Results

In this section, we report the findings of our study, focusing on the limitations of pairwise relationships for biological knowledge representation we identify. We also introduce proposals to avoid these limitations with more expressive representation models and discuss the impacts of these limitations on knowledge inference for LBD.

Our proposals focus on the two general extensions to the pairwise knowledge representation framework introduced previously: nested relationships and hypergraphs. Nested relationships provide a flexible approach for representing complex, multi-level interactions, while hypergraphs enable the modeling of higher-order interactions between more than two entities.

### Limitations of pairwise relationships

Through analysis of 56 abstracts, we identified seven types of limitations in pairwise relationships (Table 3). In the following subsections, we illustrate how these structures address specific limitations identified in the Alzheimer’s Disease corpus we study. For each limitation type, we first provide a conceptual explanation of the limitation, followed by an illustrative example of a discovery statement. The example is presented in graph format. We first construct a simple graph using only pairwise relationships and describe the limitations of that representation. We then illustrate how the more expressive structures can be adopted to address the concerns.

**Table 3:** Summary of pairwise relationship limitation types and frequency of occurrence in 56 articles. Alternative representation structures that can overcome the limitation are assigned for each limitation type. One article may have more than one limitation type

Paiwise relationship limitation type	#of abstracts	Alternative representation
Mechanism/process from a modified entity	22	Nested relationship
Modifier that adds granular information for entities	17	Nested relationship
Experimental model as a constraint	16	Nested relationship
Use of a method	12	Nested relationship
Specific context as a constraint	10	Nested relationship
Lack of context for a general concept	5	Hypergraph
More than two entities interacting together	2	Hypergraph

Each limitation type is discussed independently for clarity, even though some articles exhibit multiple limitations. In such cases, we manually isolate and address each limitation type individually to ensure a clear demonstration of how individual patterns resolve specific issues. The step-by-step process of removing each limitation type for articles with multiple limitations is detailed in the Supplementary information. Once all limitations are addressed, the resulting representation is shown as an “ideal complex graph”, which integrates the proposed representation patterns holistically. Due to space constraints, we specify the limitation type as it occurs in the most relevant component of each discovery statement (e.g., within subjects or objects). Comprehensive examples, including all affected components of the statements, are provided in the Supplementary information.

Although each example illustrates a specific limitation, these representation mechanisms provide solutions that accommodate multiple limitations, as summarized in Table 3. They offer flexible and broadly applicable solutions for advancing knowledge representation in complex biological domains.

#### Specific context as a constraint

If a finding and its context cannot be represented by a pair of nodes, a pairwise relationship is not able to indicate a relationship between a discovery and its corresponding context. This limitation, categorized in Table 3 as “Specific context as a constraint”, can be modeled with a nested relationship. Nested relationships allow for representing findings and their associated contexts as a cohesive structure, preserving both the discovery’s semantic integrity and its underlying pairwise relationships.

**Fig. 5:**
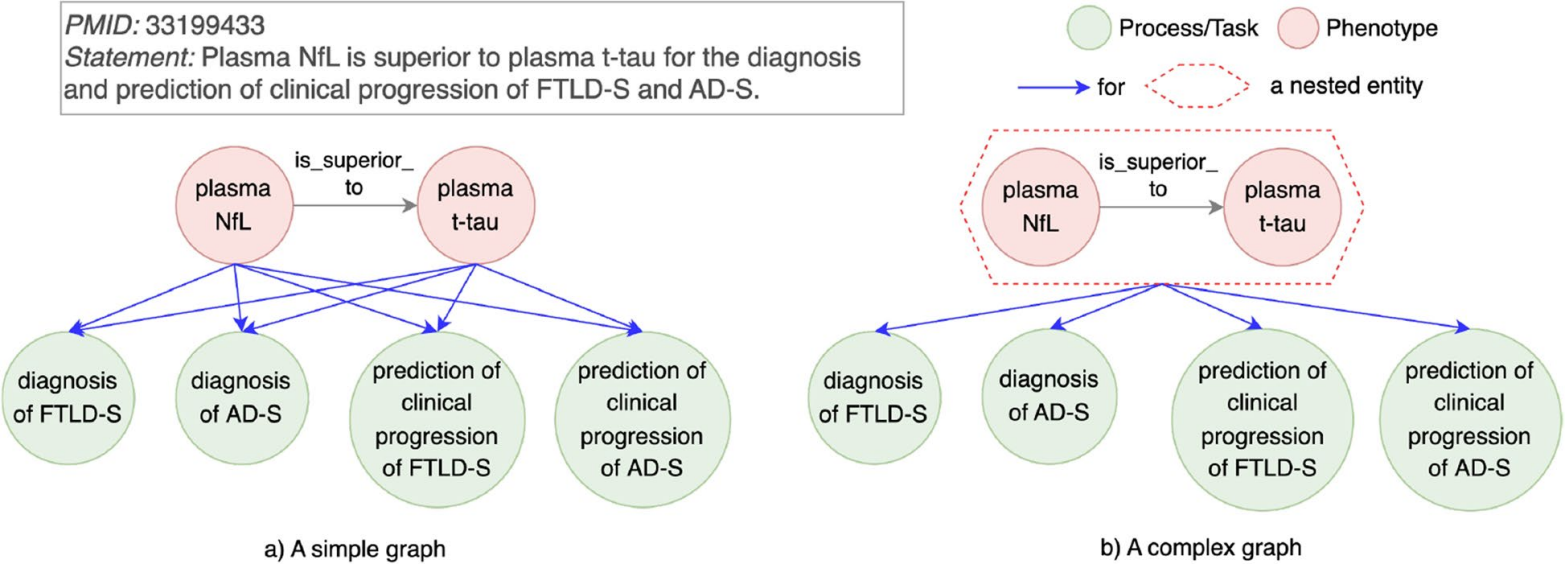
An example for a pairwise relationship limitation type “Specific context as a constraint”. Left (**a**): A set of pairwise relationships between two biomarkers (plasma NfL and plasma t-tau) and four disease diagnoses and predictions (diagnosis of FTLD-S, diagnosis of AD-S, prediction of clinical progression of FTLD-S, prediction of clinical progression of AD-S). Right (**b**): A pairwise relationship between two biomarkers plasma NfL::is superior to::plasma t-tau is grouped as an entity. Each of the four disease diagnoses and predictions is used as a context for the grouped entity

In Fig. 5, a discovery statement compares two biomarkers plasma NfL and plasma t-tau in the context of diagnosis and progression of two diseases. The finding is that plasma NfL is superior to plasma t-tau, for the purposes of diagnosis of frontotemporal lobar degeneration syndromes (FTLD-S), diagnosis of Alzheimer disease syndromes (AD-S), prediction of clinical progression of FTLD-S, and prediction of clinical progression of AD-S. In a simple graph (Fig. 5a), each of the two biomarkers is linked with each of four contexts with a predicate for. However, this representation fails to capture the discovery statement’s true meaning: a comparison between two biomarkers under four specific contexts. A nested relationship resolves this limitation by grouping the finding that (plasma NfL is superior to plasma t-tau) into a nested entity (Fig. 5b). Then, a nested entity is explicitly connected to four contexts, accurately representing the discovery as a whole. Also, the use of a nested relationship does not override the simpler pairwise relationships. For instance, the atomic pairwise relationship plasma NfL for diagnosis of AD-S remains accessible in the complex graph (Fig. 5b). These atomic relationships are inherently retained within the structure of a complex graph, where they can be detected for downstream tasks such as link prediction.

A nested relationship can represent a variety of scenarios where findings must be contextualized. In therapeutic studies, findings often depend on specific contexts, such as drug efficacy being tested under different patient populations or disease stages. Mechanistic findings often depend on environmental contexts such as temperature, pH, or specific laboratory conditions. In clinical studies, the outcomes of interventions often vary across disease subtypes.

#### Experimental model as a constraint

In Alzheimer’s Disease (AD) research, findings are often tied to specific experimental (animal) models, which provide a critical context for interpreting the results. However, when findings involve multiple nodes (e.g., entities and relationships), pairwise relationships in a simple graph fail to fully link the finding to its corresponding experimental model. This limitation is categorized in Table 3 as “Experimental model as a constraint.”

**Fig. 6:**
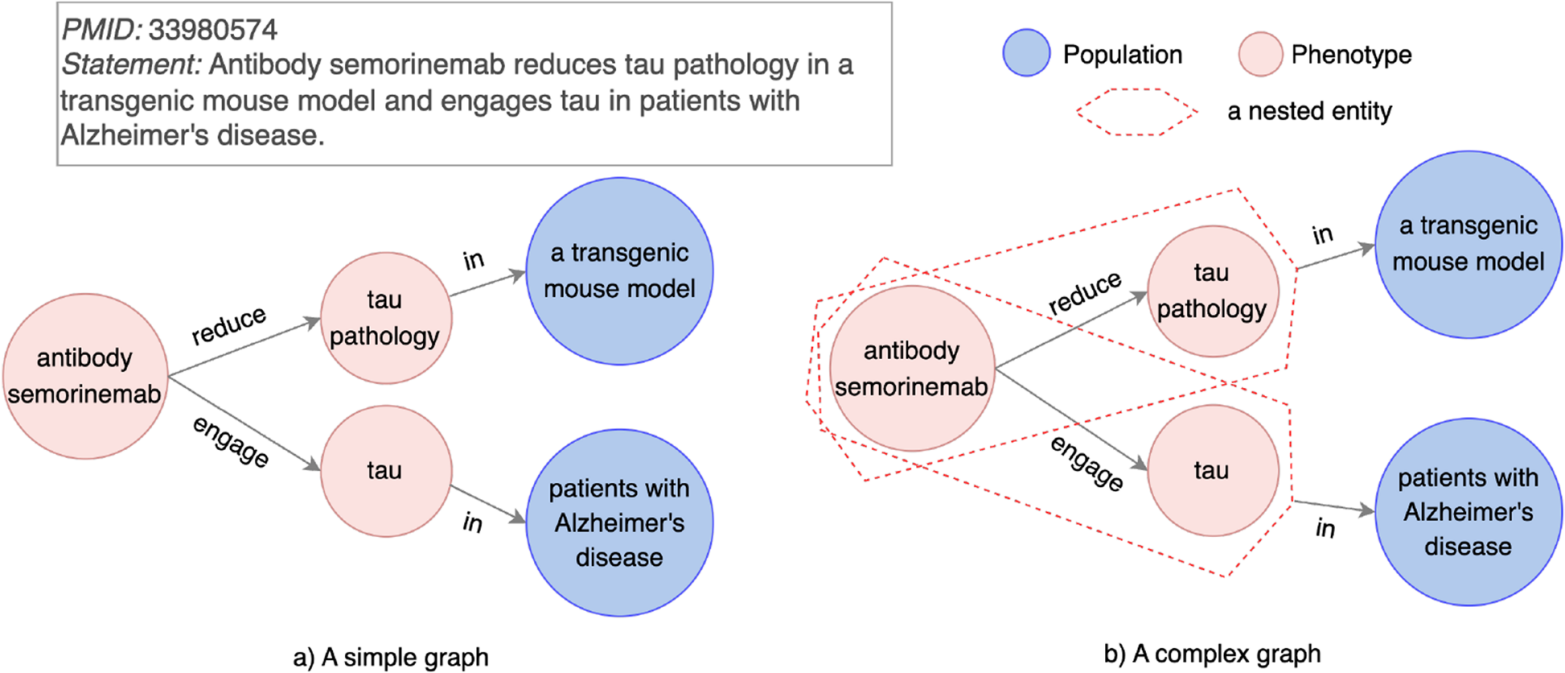
An example for a pairwise relationship limitation type “Experimental model as a constraint”. Left (**a**): Only one neurodegenerative disorder tau pathology instead of a finding antibody semorinemab reduces tau pathology is linked to an experimental model a transgenic mouse model. Only one biomarker tau instead of a finding antibody semorinemab engages tau is linked to an experimental model patients with Alzheimer’s disease. Right (**b**): Each of two findings in a graph (antibody semorinemab::reduce::tau pathology and antibody semorinemab::engage::tau) is grouped as an entity. Each grouped entity is connected to a corresponding experimental model with a relation of in

For instance, the discovery statement in PMID 33980574 (Fig. 6) is about two findings with two different experimental models. With a transgenic mouse model, antibody semorinemab reduces tau pathology. With patients with Alzheimer’s disease, antibody semorinemab engages tau. However, pairwise relationships only connect part of these findings with corresponding models – tau pathology::in::a transgenic mouse model and tau::in::patients with Alzheimer’s disease (Fig. 6a). This partial representation obscures the full discovery context.

To address this limitation, we propose the use of the nested relationship mechanism, which allows a finding to be linked to its associated experimental model(s). In this example, nested relationships represent the two findings as distinct units: antibody semorinemab::reduce::tau pathology and antibody semorinemab::engage::tau (Fig. 6b). The complex graph then conveys that:

- A finding of “antibody semorinemab reducing tau pathology” is discovered in a transgenic mouse model.
- A finding of “antibody semorinemab engaging tau” is discovered in patients with Alzheimer’s disease.

This use of nested relationships ensures that experimental models are accurately contextualized within the graph, preserving the integrity of discovery statements. The approach generalizes to other scenarios where findings are tied to experimental models. In preclinical animal studies, research often involves multiple models, such as mice and non-human primates. Alternatively, the same type of model can be divided into experimental groups treated with varying proportions or durations of interventions. For example, in PMID 36840284, rats were divided into four groups with different treatments: 1) A control group was treated orally with the vehicle for 30 days and given four injections of saline. 2) An LPS-induced group was treated with the vehicle for 30 days and given four injections of LPS. 3) A test group was treated with MASE 200 mg/kg for 30 days and given four injections of LPS. 4) Another test group was treated with MASE 400 mg/kg for 30 days and given four injections of LPS. Nested relationships can represent each experimental group and its associated treatment context independently, preserving the experimental distinction for downstream analysis.

In clinical research, findings are often associated with specific trial phases, such as Phase I and Phase II. Mechanistic studies often compare findings in in vitro systems (e.g., cell cultures) to those in in vivo animal models. All these scenarios require preserving distinct models for findings. By grouping findings and their associated experimental models into cohesive units, use of a nested relationship ensures that contextual meanings of findings are kept.

#### Use of a method

In some discovery statements, a method is central to reveal a finding. However, when a finding consists of multiple entities and relationships, pairwise relationships often fail to connect a method to the entire finding, leading to incomplete representations. This limitation is categorized in Table 3 as “Use of a method”.

**Fig. 7:**
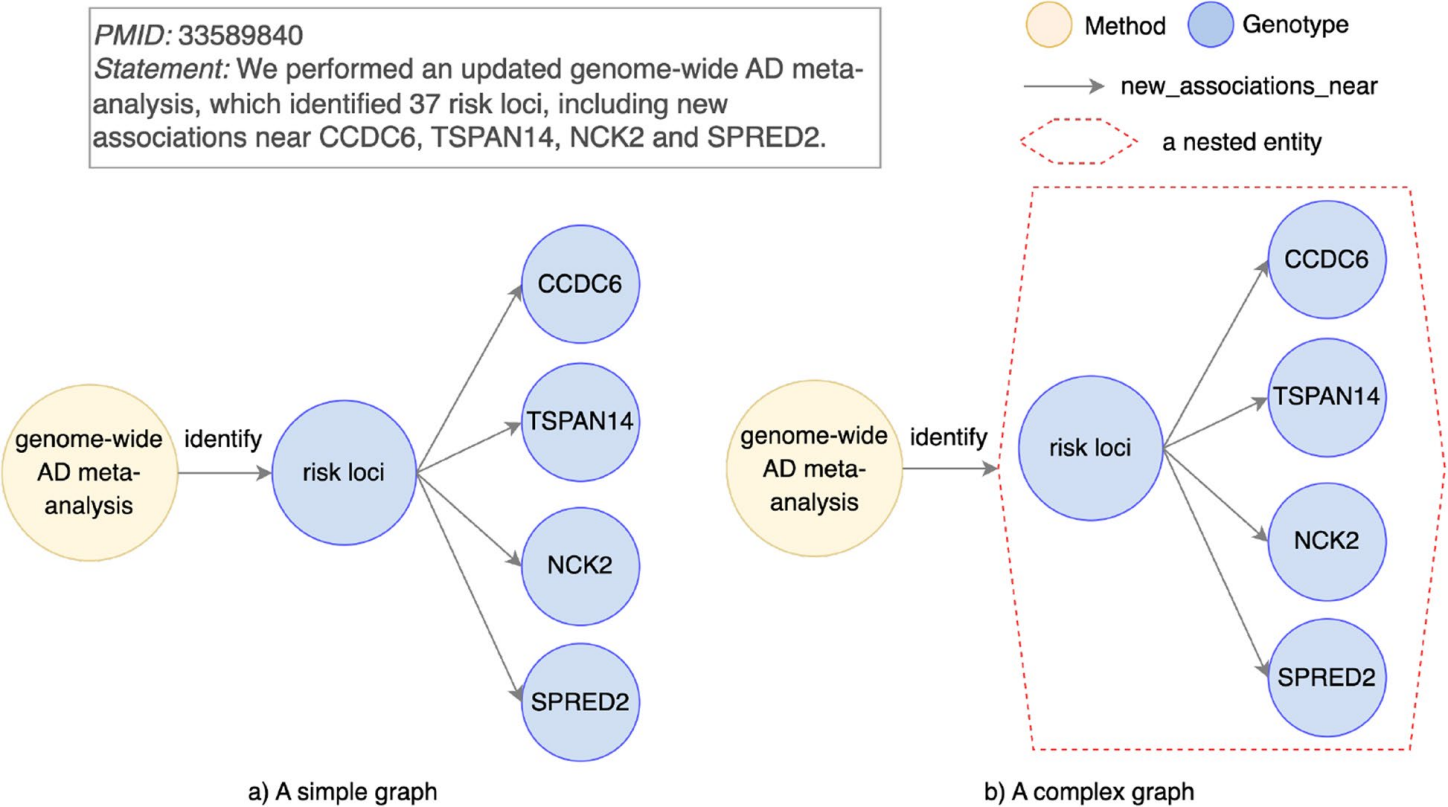
An example for a pairwise relationship limitation type “Use of a method”. Left (**a**): A method genome-wide AD meta-analysis is connected to part of a finding risk loci with a predicate identify. Right (**b**): A method genome-wide AD meta-analysis is connected to a complete finding of risk loci including new associations near CCDC6, TSPAN14, NCK2 and SPRED2. The complete finding is represented as a nested entity

For instance, PMID 33589840 (Fig. 7), a discovery statement shows that a method genome-wide AD meta-analysis is used to identify risk loci, which includes new associations near four genes CCDC6, TSPAN14, NCK2, and SPRED2. In a simple graph (Fig. 7a), genome-wide AD meta-analysis is only pointed to part of a finding risk loci, while new associations with four genes are left unconnected. This fragmented representation fails to reflect the complete discovery statement.

To address this limitation, we again draw on nested relationships. In this example, a nested entity groups risk loci and four associated genes (CCDC6, TSPAN14, NCK2, and SPRED2). This relationship is then linked to the method genome-wide AD meta-analysis as a unit (Fig. 7b). The resulting complex graph fully aligns with the discovery, representing the method and its complete findings as a unified structure.

A nested relationship is a general solution for representing method-driven discoveries where methods interact with multiple entities or relationships. For instance, in biomarker studies, methods like machine learning models or statistical tests can be linked to multiple entities (e.g., a set of features or predictors) to show their role in generating predictive results. In experimental procedures, methods such as mass spectrometry or immunohistochemistry can be explicitly connected to groups of findings, such as identifying multiple proteins or pathways.

#### Mechanism/process from a modified entity

Some entities in discovery statements become a mechanism or a process through modification by another entity (i.e., a modifier) to acquire additional attributes, such as temporal details. Pairwise relationships fail to illustrate a nested entity constructed from an entity modifying another entity, when the nested entity needs to be linked to other nodes. This limitation is categorized in Table 3 as “Mechanism/process from a modified entity”.

**Fig. 8:**
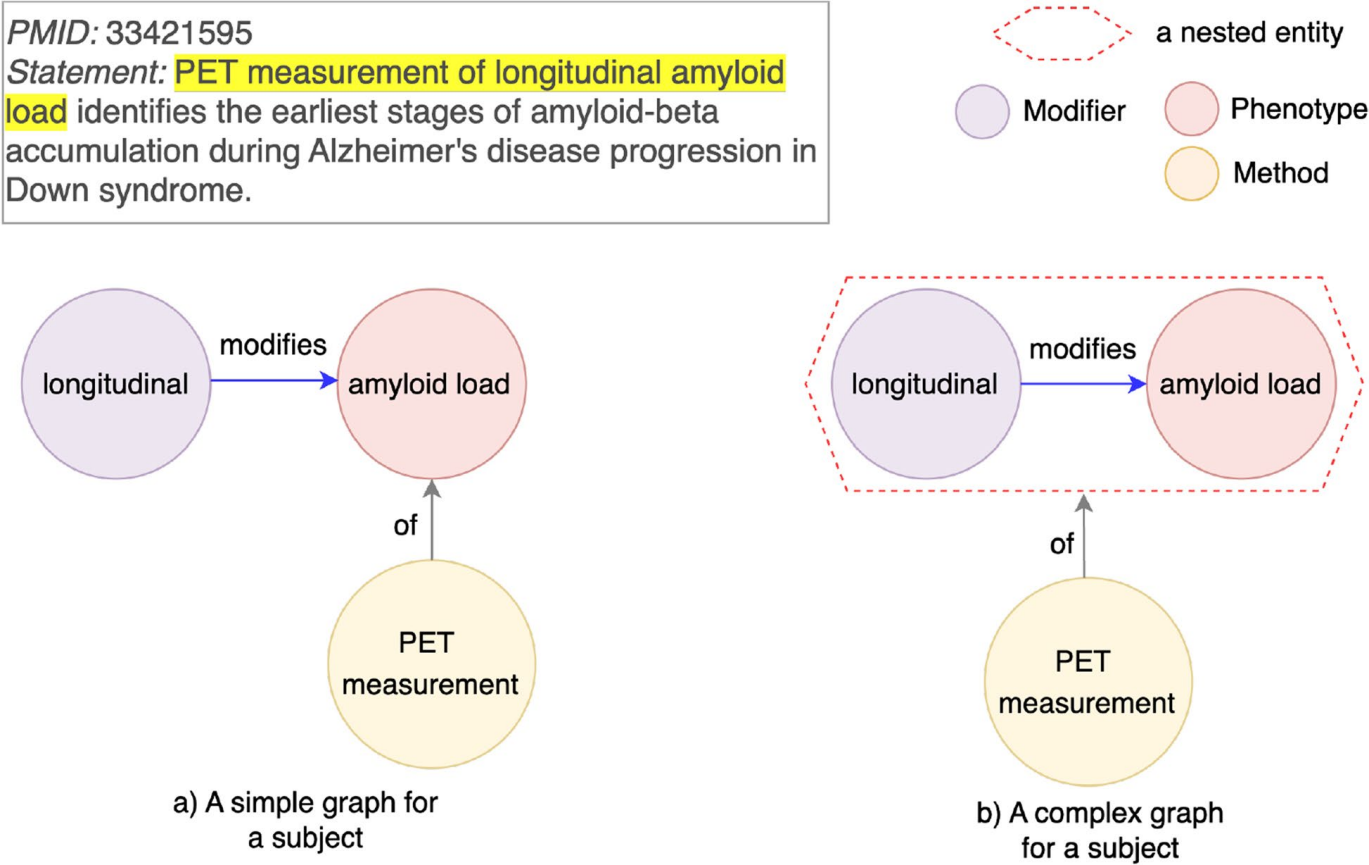
An example for a pairwise relationship limitation type “Mechanism/process from a modified entity”. Left (**a**): Pairwise relationships between a modifier longitudinal and a modified entity amyloid load. Right (**b**): A modifier and a modified entity grouped as a process longitudinal amyloid load

For example, in the highlighted statement derived from PMID 33421595 (Fig. 8), Longitudinal amyloid load is a process of measuring amyloid load in the long term. A chronological record of amyloid load is used to identify patients with Down syndrome at risk for Alzheimer’s Disease. The term longitudinal is a modifier for an entity amyloid load. A simple graph (Fig. 8a) only states two separate discoveries: 1) longitudinal::modify::amyloid load, and 2) PET measurement::of::amyloid load. These two disconnected relationships do not convey the intended usage of PET measurement to assess the longitudinal amyloid load.

To address this limitation, we apply nested relationships to group together amyloid load and its modifier longitudinal. Then, the nested entity longitudinal amyloid load is linked to the PET measurement (Fig. 8b). This representation accurately conveys that PET measurement applies specifically to longitudinal amyloid load, preserving the full semantics of the discovery statement.

The benefits of knowledge representation with nested relationships generalizes beyond this example. Modifiers such as accumulation can transform entities like amyloid-beta into processes (e.g., amyloid-beta accumulation), which can then be linked to Alzheimer’s Disease progression or other nodes (Appendix 3).

#### Modifier that adds granular information for entities

In some discovery statements, modifiers are used to provide additional granularity to an entity without altering its fundamental type. Unlike transformations that create mechanisms or processes (as discussed in “Mechanism/process from a modified entity” section), these modifiers add descriptive attributes to refine the meaning of the entity. When a modifier and its associated entity cannot be represented as a single node, a simple graph often omits the modifier, leading to an incomplete or inaccurate representation of the discovery. This limitation is categorized in Table 3 as “Modifier that adds granular information for entities”.

**Fig. 9:**
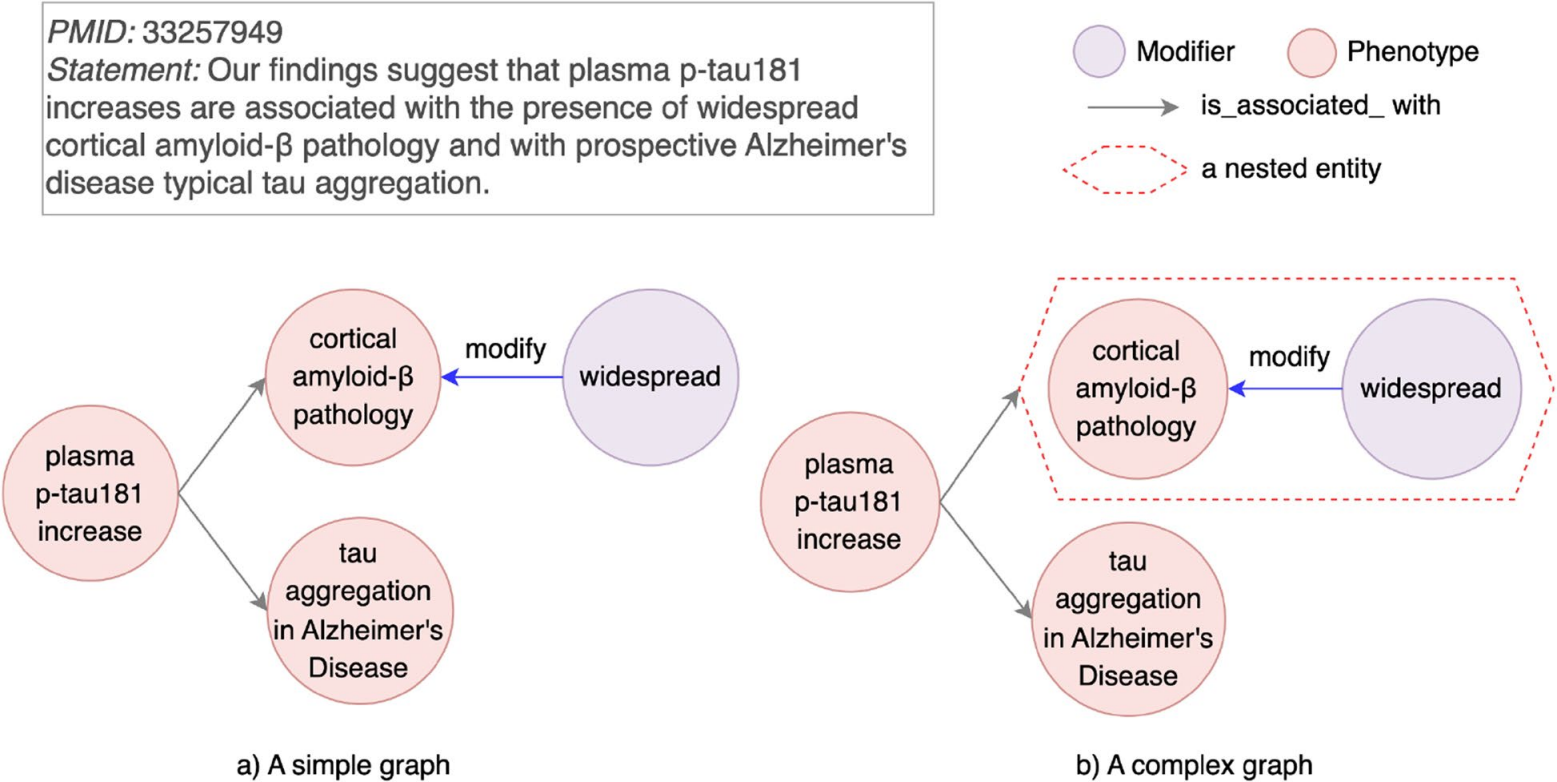
An example for a pairwise relationship limitation type “Modifier that adds granular information for entities”. Left (**a**): A modifier widespread and a corresponding genotype cortical amyloid-$\beta$ pathology are connected with a relation of modify. An entity plasma p-tau181 increase is associated with an entity cortical amyloid-$\beta$ pathology only. Right (**b**): widespread pointing to cortical amyloid-$\beta$ pathology with a relation of modify is grouped as a nested entity. plasma p-tau181 increase is associated with a granular entity. To avoid confusion for another limitation type, we fix the limitation type of “‘Mechanism/process from a modified entity” for the entity “plasma p-tau 181 increase”, which is a nested entity by grouping “plasma p-tau 181” and “increase”

For instance, in PMID 33257949 (Fig. 9), a discovery statement describes how plasma p-tau181 increases are associated with widespread cortical amyloid-$\beta$ pathology. The modifier *widespread* provides a spatial attribute to the entity cortical amyloid-$\beta$ pathology, which refines its meaning. In a simple graph (Fig. 9a), widespread points to cortical amyloid-$\beta$ pathology with a predicate modify. It is only the entity cortical amyloid-$\beta$ pathology that is directly linked to plasma p-tau181 increases. This representation fails to capture the granularity provided by the modifier *widespread*.

To address this limitation, we apply a nested relationship. In this example, widespread and cortical amyloid-$\beta$ pathology are grouped into a single nested entity as widespread cortical amyloid-$\beta$ pathology (Fig. 9b). This nested entity is then linked to plasma p-tau181 increases, preserving the complete semantic detail of the discovery statement.

Beyond this example, the nested relationship approach generalizes to other scenarios where modifiers add granularity to entities. For instance, elevated plasma amyloid-$\beta$ (PMID 17620492) combines a modifier *elevated* with plasma amyloid-$\beta$ to provide quantitative refinement. Similarly, diffuse amyloid-$\beta$ plaques (PMID 30040735) reflects spatial characteristics added by a modifier *diffuse*. Nested relationships ensure these granular details are accurately represented, providing a flexible solution for this limitation.

#### Lack of context for a general concept

In some discovery statements, general concepts are central to knowledge being conveyed, but their meaning is dependent on specific contexts. Without explicitly linking these general concepts to their contexts, it is hard to represent the full semantic meaning of the discovery statement. This limitation is categorized in Table 3 as “Lack of context for a general concept”.

**Fig. 10:**
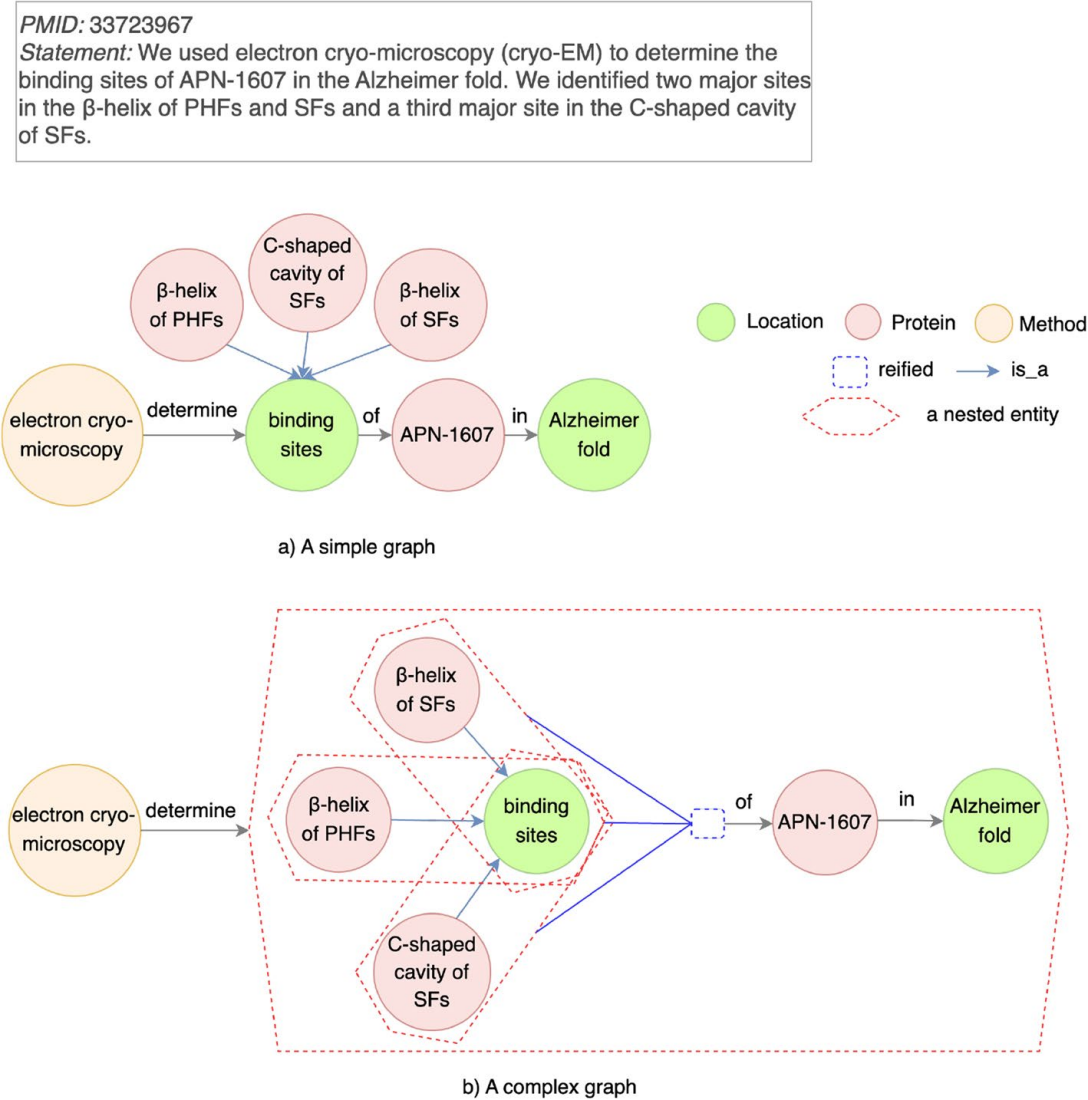
An example for a pairwise relationship limitation type “Lack of context for a general concept”. Top left (**a**): Each of three major sites ($\beta$-helix of PHFs, $\beta$-helix of SFs, and C-shaped cavity of SFs) is connected to a general concept binding sites with an is_a relation. binding sites is an object determined by a tool electron cryo-microscopy. Bottom (**b**): A complex graph that removes a limitation of “Lack of context for a general concept”. A general concept binding sites and its contexts for three major sites are reified as an entity

For example, in Fig. 10, a discovery statement describes binding sites as a general concept contextualized by three specific sites: $\beta$-helix of paired helical filaments (PHFs), $\beta$-helix of straight filaments (SFs), and C-shaped cavity of SFs. These three major sites are the specific contexts in which binding sites are discovered for APN-1607 in the Alzheimer fold. A simple graph (Fig. 10a) only links binding sites to three major sites using an “is_a” relationship, but this representation does not capture how three sites provide a necessary context for interpreting binding sites in the Alzheimer fold.

To address this limitation, we employ a hypergraph, which groups the general concept (binding sites) and its associated contexts ($\beta$-helix of PHFs, $\beta$-helix of SFs, and C-shaped cavity of SFs) into a single cohesive structure. In the hypergraph representation (Fig. 10b), an interaction between binding sites and its three major sites is explicitly represented, capturing the full contextual meaning. This structure incorporates an overarching discovery, which links the interaction to APN-1607 in the Alzheimer fold. The removal of other limitation types in this example is shown in Appendix 1.

Beyond this example, hypergraphs generalize to other cases where general concepts require contextual relationships. For instance, a general concept biomarker candidate might depend on specific disease contexts such as mild cognitive impairment or early-onset Alzheimer’s Disease. Similarly, a general concept drug mechanism may depend on contextual links to specific pathways or target proteins. Hypergraphs enable these relationships to be expressed explicitly, preserving the semantic meaning of general concepts in their appropriate contexts.

#### More than two entities interacting together

When more than two entities interact with each other, the interaction itself rather than an individual entity may be the most relevant actor in a knowledge discovery. Pairwise relationships fail to capture the holistic nature of such interactions, as they only represent individual pairwise connections between entities, leading to an incomplete representation. This limitation is categorized in Table 3 as “More than two entities interacting together”.

**Fig. 11:**
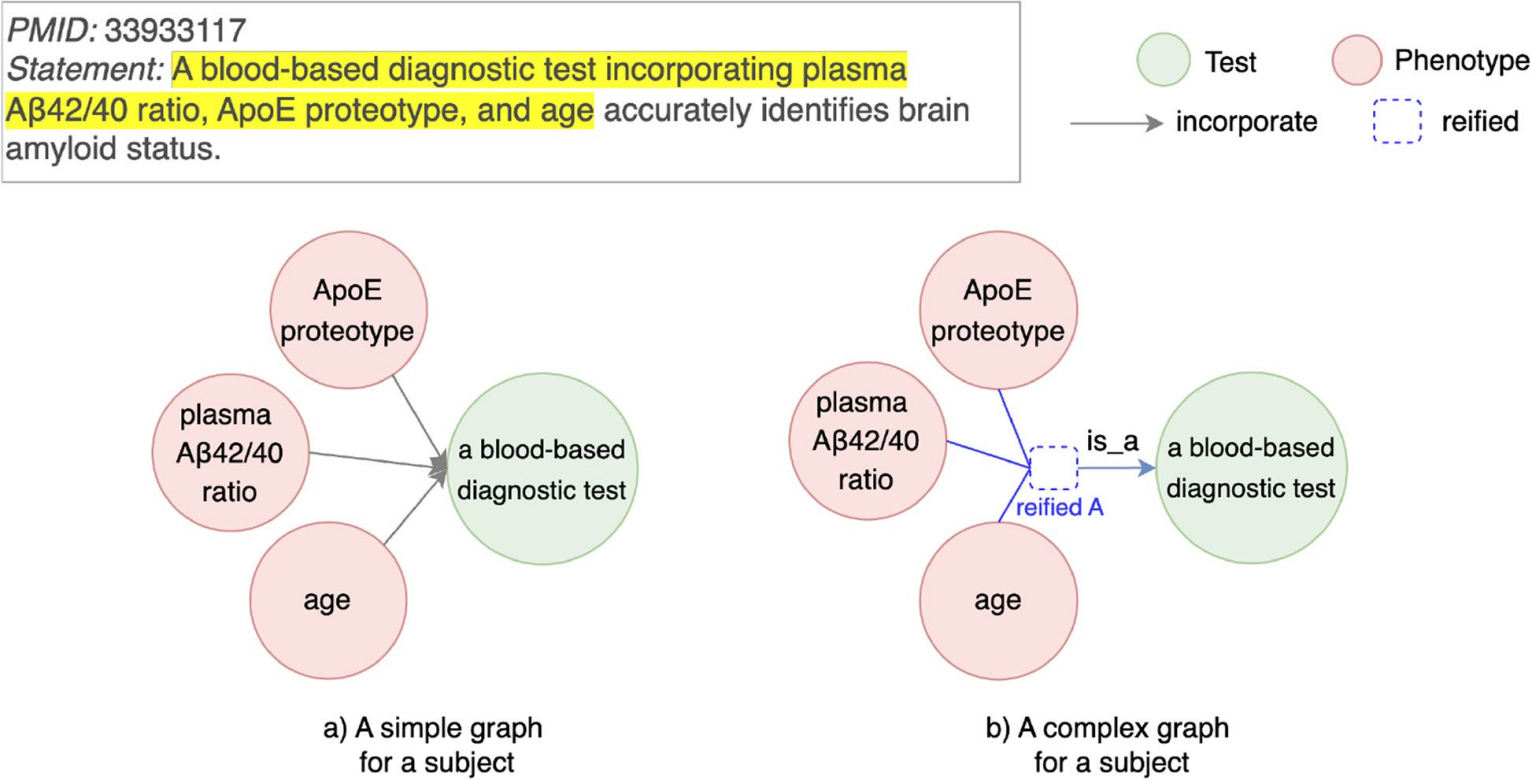
An example for a pairwise relationship limitation type “More than two entities interacting together”. Left (**a**): Each of three biomarkers (plasma A$\beta$42/40 ratio, ApoE proteotype, and age) is connected to a general concept a blood-based diagnostic test with a relation of incorporate. a blood-based diagnostic test is a subject that identifies brain amyloid status. Right (**b**): One limitation of “More than two entities interacting together” is removed with a hypergraph. Three biomarkers are reified as an entity (denoted as reified A). A reified biomarker is linked with a general concept a blood-based diagnostic test with a relation of is_a

For instance, in PMID 33933117 (Fig. 11), a blood-based diagnostic test is developed by a combination of three biomarkers: plasma A$\beta$42/40 ratio, ApoE proteotype, and age. This combination is used to identify brain amyloid status. In a simple graph (Fig. 11a), pairwise relationships represent each of these three biomarkers as individually incorporated into the test. This representation fails to capture the collective interaction among all three biomarkers that comprises the test.

To address this limitation, we apply hypergraphs, which represents an interaction among multiple entities in a single structure. In this example, an interaction among three biomarkers – plasma A$\beta$42/40 ratio, ApoE proteotype, and age – are grouped together as a node reified A (Fig. 11b), representing their collective role as a diagnostic test. The removal of other limitation types in this example is shown in Appendix 2.

Beyond this example, hypergraphs generalize to other scenarios where interactions among multiple entities are essential under a biomedical context. In drug discovery, a combination of multiple drugs may work together to treat a disease. A hypergraph can represent an interaction among these drugs as a cohesive unit, and then link a drug combination to a treatment outcome or a disease symptom. Similarly, a protein complex involving several proteins can be reified as a single entity, which can further be linked to a biological function.

### Impacts of pairwise relationship limitations on an LBD task

The previous Section shows that the choice of representation framework affects how a knowledge graph is constructed for biological discovery statements. In this section, we explore knowledge inference in LBD as a downstream task utilizing knowledge graphs, further showing the influence of representation frameworks. To illustrate these impacts, we provide three illustrative case studies, each demonstrating how limitations in pairwise relationships affect the inferences that can be drawn from knowledge graphs. As shown in Table 4, we group impacts of pairwise relationship limitations on knowledge inference into three levels of increasing severity:

- Experimentally infeasible hypotheses: Hypotheses that lack critical contextual or biological details, making them impractical to test in laboratory experiments.
- Literature-inconsistent hypotheses: Hypotheses that are experimentally feasible but misalign with known discoveries in the literature, leading to incorrect experimental designs and wasted resources.
- Oversimplified hypotheses explanations: Hypotheses whose explanations lack sufficient details, limiting their interpretability despite being experimentally feasible and potentially successful.

We further assess impacts on knowledge inference with three aspects: experiment feasibility, experiment success, and explanation granularity. “Experiment feasibility” indicates whether an experiment is feasible to be carried out with the generated hypothesis. “Experiment success” refers to whether an experiment will yield successful results, aligned with known biological mechanisms. “Explanation granularity” captures whether the explanation for the generated hypothesis contains sufficient detail to be scientifically meaningful.

In the subsections below, we provide conceptual illustrative case studies to demonstrate how pairwise relationships fall short in these areas and how alternative representation approaches, such as nested relationships and hypergraphs, address these limitations. These case studies serve as thought experiments, not experimental results, and focus on conceptual comparisons rather than real-world data. Future work could implement these more expressive representations in real AD-LBD systems to evaluate their impact on knowledge inference tasks.

**Table 4:** Impacts on knowledge inference with different pairwise relationship limitation types and aspects of impacts (*EF: Experiment Feasibility, ES: Experiment Success, EG: Experiment Granularity*)

*Impacts on knowledge inference*	*Pairwise relationship limitation type(s)*	*EF*	*ES*	*EG*
Experimentally infeasible hypotheses	Lack of context for a general concept	✗	✗	✗
Literature-inconsistent hypotheses	More than two entities interacting together Experimental model as a constraint Use of a method Specific context as a constraint Mechanism/process from a modified entity	✓	✗	✗
Oversimplified hypotheses explanations	Modifier that adds granular information for entities	✓	✓	✗

#### Experimentally infeasible hypotheses

**Fig. 12:**
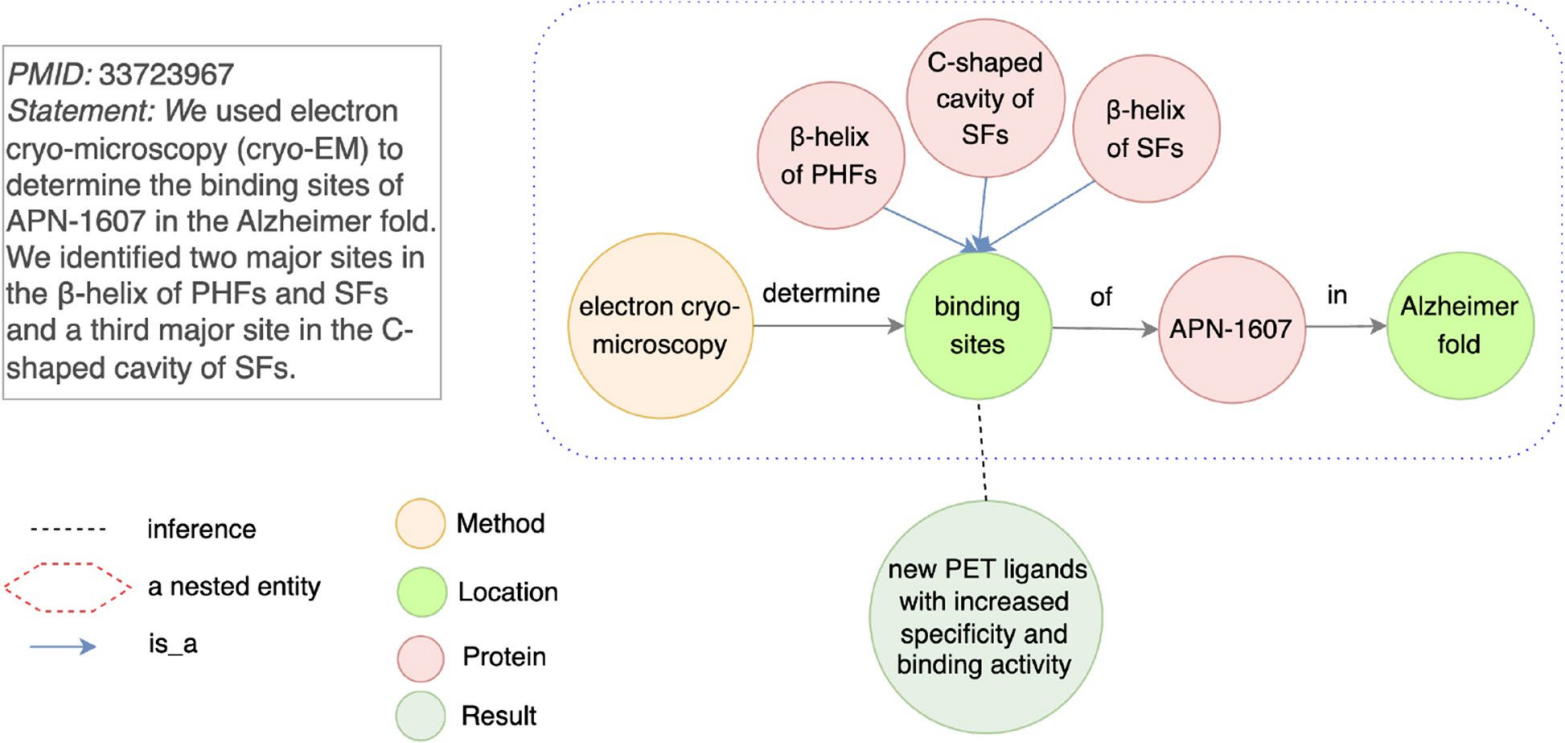
An example for an impact as “Experimentally infeasible hypotheses”. A knowledge graph with pairwise relationship limitation type “Lack of context for a general concept” is constructed from PMID 33723967. Knowledge inference is performed on a node new PET ligands with increased specificity and binding activity and a general node binding sites without contexts

The most detrimental impact on knowledge inference is the generation of hypotheses that cannot be practically tested through laboratory experiments to study AD diagnosis, treatment, and molecular mechanisms. The pairwise relationship limitation type “Lack of context for a general concept” has such a negative impact. Figure 12 shows a simple graph, where a general concept binding sites does not have a context for three major sites ($\beta$-helix of PHFs, $\beta$-helix of SFs, and C-shaped cavity of SFs). If a new link is inferred between a node new PET ligands with increased specificity and binding activity and a general node binding sites, the generated hypothesis is not applicable in a lab. What a general concept binding sites means is not stated. It is the three major sites – $\beta$-helix of PHFs, $\beta$-helix of SFs, and C-shaped cavity of SFs – that have biological meaning in designing new PET ligands with increased specificity and binding activity.

#### Literature-inconsistent hypotheses

**Fig. 13:**
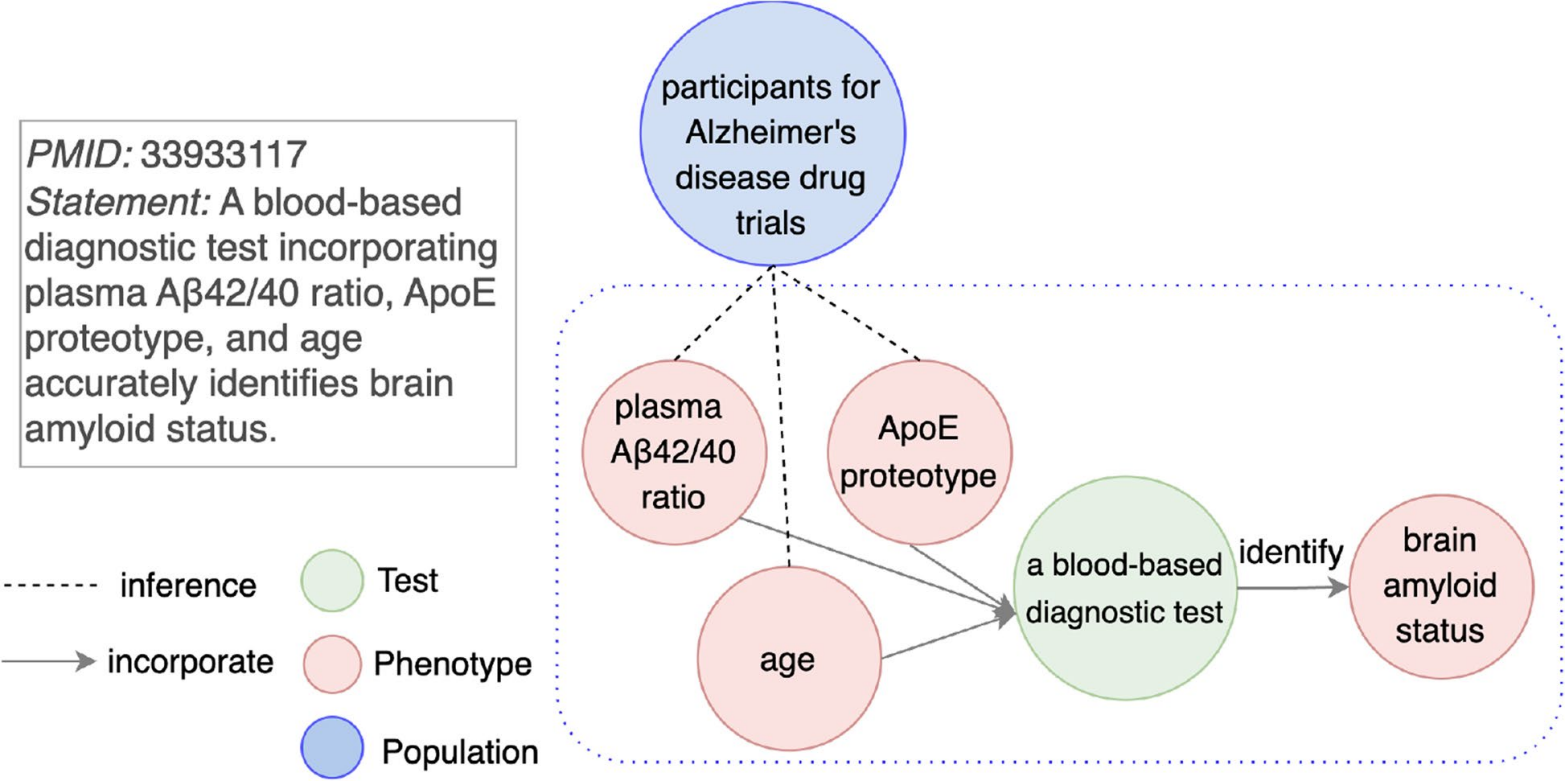
An example for an impact as “Literature-inconsistent hypotheses”. A knowledge graph with pairwise relationship limitation type “More than two entities interacting together” is constructed from PMID 33933117. Knowledge inference is performed on a node participants for Alzheimer’s disease drug trials and any of three nodes (plasma A$\beta$42/40 ratio, ApoE proteotype, and age). All of these three hypotheses may be inaccurate because the discovery statement is about combining three biomarkers as a blood-based diagnostic test

Another impact on knowledge inference in LBD is the generation of literature-inconsistent hypotheses. Although a hypothesis may be experimentally feasible, it does not accurately reflect the knowledge presented in the AD literature. The pairwise relationship limitation type “More than two entities interacting together” has such an impact. In PMID 33933117 (Fig. 13), a discovery statement is about using three biomarkers together (plasma A$\beta$42/40 ratio, ApoE proteotype, and age) as a blood-based diagnostic test. Such a combination is able to identify brain amyloid status. However, with pairwise relationships only, each of the three biomarkers appears to independently identify brain amyloid status. A relation of combination among three biomarkers is not reflected. Suppose a new node participants for Alzheimer’s disease drug trials is inferred with any of the three biomarkers, a generated hypothesis is inaccurate compared to the statement. Using ApoE proteotype as a standard to enroll participants for Alzheimer’s disease drug trials may not work. A hypothesis that aligns with AD literature is to use all three biomarkers ApoE proteotype, plasma A$\beta$42/40 ratio, and age together to enroll participants for Alzheimer’s disease drug trials. Experimenting with an inaccurate hypothesis wastes resources for domain experts.

#### Oversimplified hypotheses explanations

**Fig. 14:**
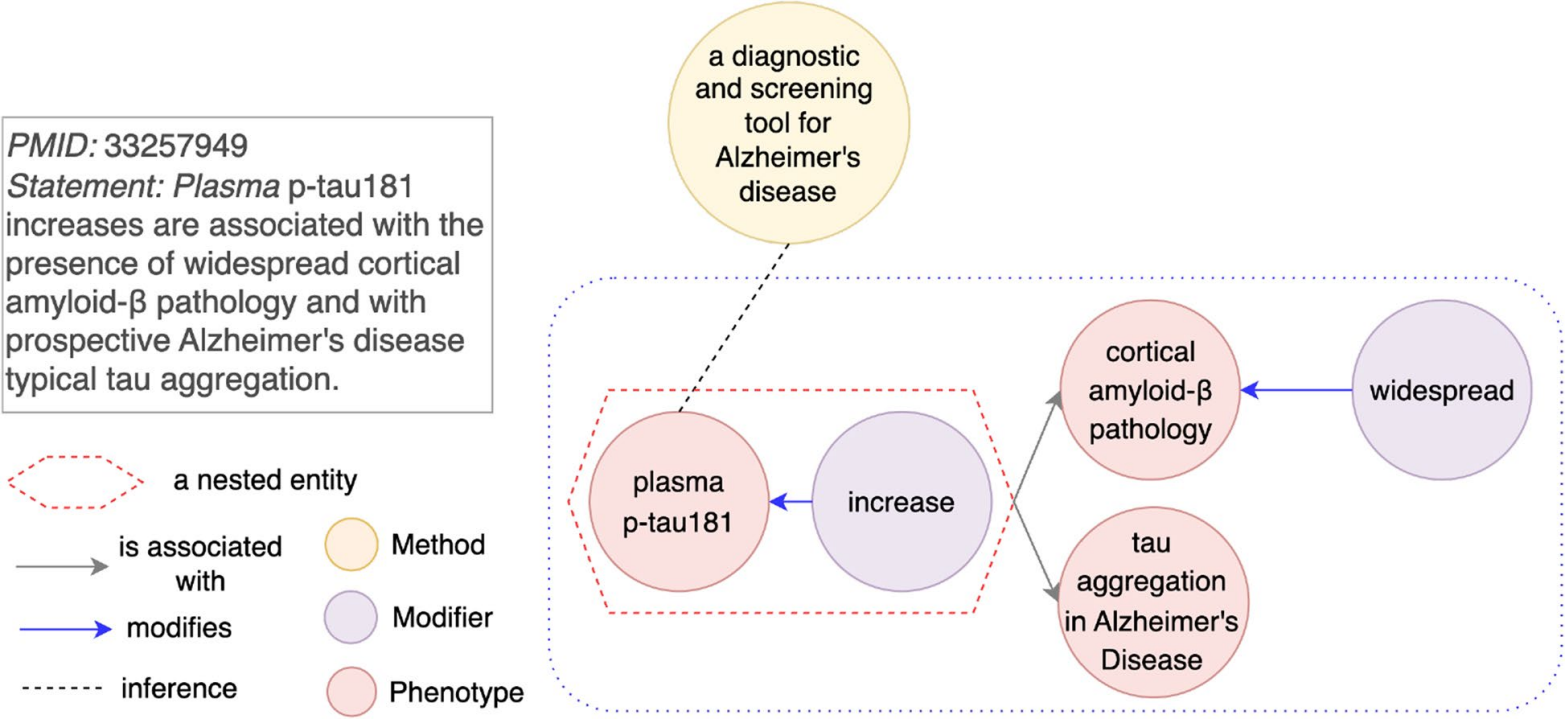
An example for an impact as “Oversimplified hypotheses explanations”. A knowledge graph with pairwise relationship limitation type “Modifier that adds granular information for entities” is constructed from PMID 33257949. Knowledge inference is performed on a node a diagnostic and screening tool for Alzheimer’s disease and a node plasma p-tau181. An explanation for the inference as plasma p-tau181 increase associated with cortical amyloid-$\beta$ is not as refined as an explanation of plasma p-tau181 increase associated with widespread cortical amyloid-$\beta$

Another impact on knowledge inference is the generation of hypotheses explanations with insufficient details from discoveries in AD literature, because the explanations lack necessary modifiers. However, such impacts are less severe in terms of experimental feasibility and success compared to “Literature-inconsistent hypotheses” (“Literature-inconsistent hypotheses” section). With the generated hypothesis, an experiment is not only feasible to be conducted, but it also has a higher chance of being successful. The primary deficit is in explaining the hypothesis. If modifiers can be nested with entities, explanations for generated hypotheses will be more granular and aligned with the literature. As shown in Fig. 14, statements in PMID 33257949 show plasma p-tau181 increase being associated with widespread cortical amyloid-$\beta$. However, in a simple graph, plasma p-tau181 increase is connected with cortical amyloid-$\beta$ without the modifier *widespread*. Without representation of more granular information, when a new node a diagnostic and screening tool for Alzheimer’s disease is inferred with the blood biomarker plasma p-tau181, an explanation for this inference becomes plasma p-tau181 increase associated with cortical amyloid-$\beta$. Lacking of a modifier *widespread* makes the explanation oversimplified for cortical amyloid-$\beta$.

## Discussion

### Implications of the findings

#### Link prediction for extended representations

While the primary focus of this study is on exploring alternative representations for constructing knowledge graphs, it is worth discussing the implications of these representations in the context of link prediction. Link prediction is a standard inference method for knowledge graphs that aims to infer missing links or relationships in a knowledge graph and plays a key role in Literature-Based Discovery (LBD) systems [22]. Existing link prediction models, such as Common Neighbors (CN, [40]), are primarily designed for pairwise relationships. However, the adoption of representation structures such as nested relationships and hypergraphs introduces new challenges. For hypergraphs, standard link prediction models must be adapted to accommodate higher-order interactions. For instance, Common Neighbors can be generalized to a hyperlink prediction scenario in a hypergraph by averaging pairwise CN indices with each hyperlink [7]. Additionally, new methods that were specifically designed for hyperlink prediction in hypergraphs have been proposed, such as Hyperlink Prediction Using Resource Allocation [17]. To apply standard deep learning methods such as Node2Vec with Single-layer Perceptron on hypergraphs, hypergraph expansion methods such as clique expansion, star expansion, and line graph are required [37, 39].

While this study does not train or evaluate a link prediction system, one implication of our findings is the need to adapt standard link prediction methods and create new link prediction methods tailored to more complex knowledge graphs. Methods designed specifically for nested relationships or hypergraphs may enhance the predictive power of LBD systems.

#### Knowledge representation components

In this study, we argue that relying solely on pairwise relationships has limitations for biological knowledge representation. As a downstream task, knowledge inference in LBD shows 3 types of negative impacts from using pairwise relationships only. While alternative knowledge representation strategies can mitigate these limitations, they do not replace the need for pairwise relationships. An ideal representation involves integrating both pairwise relationships and more expressive representation structures, such as hypergraphs and nested relationships. Here we further examine components of knowledge representation for discovery statements in the corpus.

**Fig. 15:**
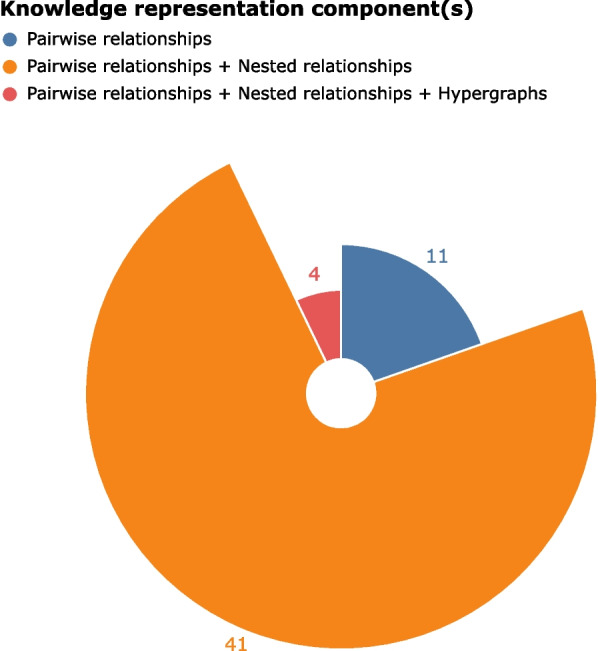
Statistics of knowledge representation components for statements

As shown in Fig. 15, 11 of 56 (around 20%) statements in our corpus are perfectly represented with pairwise relationships only. Knowledge discoveries in 45 of 56 (around 80%) articles require more than pairwise relationships only. Around 73% statements are ideally represented by a combination of pairwise relationships and nested relationships. The remaining 7% are ideally represented by all three types of knowledge representation. Pairwise relationships are essential for biological knowledge representation, allowing for co-occurrences and semantic triples. Nested relationships enhance this foundation by incorporating nested entities, while hypergraphs further extend it by capturing collective interactions among entities. Nested relationships and hypergraphs support and complement the core functionality of pairwise relationships, allowing for ideal representations for complex knowledge discoveries.

Although this study focuses on seven types of limitations observed in this dataset, we recognize that a broader dataset might reveal additional limitations. Nonetheless, the nested relationship and hypergraph mechanisms offer significant flexibility and scalability for representing biological processes. Future work is needed to validate the sufficiency of these approaches across other corpora and domains.

#### Commonalities in pairwise relationship limitations

“Limitations of pairwise relationships” section reports five types of pairwise relationship limitations that can be mitigated with nested relationships. These five limitations are categorized based on AD statement structure. These statements share certain linguistic characteristics, which relate to the need for more sophisticated knowledge representation.

Three limitation types (“Specific context as a constraint”, “Experimental model as a constraint”, and “Use of a method”) have in common that a subject and/or an object of a discovery statement comprises multiple entities, as shown in the examples above. Given this, the proposed knowledge representation solutions for these three limitations are the same: group findings as a nested entity to capture the relevant hierarchical structure.

For limitations of “Mechanism/process from a modified entity” and “Modifier that adds granular information for entities”, a shared pattern lies in the use of modifiers to create hierarchical compositions. These modifiers group multiple entities into mechanisms, processes, or granular entities, all best represented via the same nested relationship mechanism.

Identifying these patterns has practical implications for advancing the proposed knowledge representation framework. For instance, manual knowledge representation annotation may be sped up through recognition of recurring linguistic structures.

### Related knowledge representation approaches

#### BEL for information integration

Biological Expression Language (BEL) provides a structured foundation for integrating knowledge across multiple articles. In this study, we explore each article with one separate graph. To conduct downstream tasks such as link prediction, individual graphs need to be integrated into a comprehensive and interconnected knowledge graph spanning multiple articles. However, using graph formats for an integrated graph is difficult. Unlike a standard knowledge graph (e.g., with co-occurrences), where nodes from separate articles can be directly inserted into the graph, nested relationships and hypergraphs involve more complex hierarchical relationships and higher-order interactions. By encoding each discovery statement as a BEL statement, it is possible to represent complex graphs in a text-based format that can scale efficiently. A prior study [15] used BEL to construct cause-and-effect models in Alzheimer’s disease, encoding relationships between biomolecules, pathways, and clinical outcomes in scale. However, our study requires BEL to support hierarchical structures and higher-order interactions, which is more than causal relationships in the previous study. One future work is to use BEL statements on a scale to represent discovery statements both separately and collectively for nested relationships and hypergraphs.

Recent developments of Large Language Models (such as GPT-3 [4], LLaMA [32], and LLaMA 2 [33]) in the Natural Language Processing field may help with tasks including statement extraction, entity extraction, and relation extraction. These tasks are essential components for knowledge graph construction and BEL statement building. However, Large Language Models may not work perfectly, such as having a cost for removing False Positives [23] and experiencing hallucinations for fewer shots [16] in biomedical information extraction tasks.

#### Ontology-driven knowledge graph

Ontologies enable consistent and structured exchange of information for a domain by providing an agreed vocabulary and semantic framework [28]. They play an important role in biomedical knowledge representation, such as providing structured vocabularies for standardizing entities and relationships, as well as providing a conceptual framework for knowledge graph construction. Integrating ontologies into knowledge representation framework can be valuable. For instance, normalizing text entities to ontology identifiers in resources such as ChEBI^6^ (Chemical Entities of Biological Interest) implies that free-text entities “amyloid-beta” and “beta-amyloid” can be represented by a single node corresponding to the unique identifier “CHEBI:64645”. The mapping allows for consistency and interoperability among different namings of the same entity from multiple sources.

For ontologies with hierarchical structure (e.g., Gene Ontology^7^), entities and relationships can be classified into specific categories, such as “Alzheimer’s Disease being a Subtype of Neurodegenerative Diseases”. This hierarchical organization facilitates querying based on conceptual relationships. Rules and constraints in an ontology also define how entities and relationships can interact, such as “Protein participates in Pathway” and “Pathway causes or contributes to Disease” [5]. These rules and constraints support knowledge-based reasoning over a knowledge graph, such as inferring new relationships “Protein causes or contributes to Disease”. Callahan et al. [5] provide a comprehensive example of ontology-driven knowledge graph construction. The proposed PheKnowLator Ecosystem shows how ontologies play a key role at multiple stages, including mapping identifiers for data processing, aligning entity relationships to ontology hierarchies, and customizing knowledge representations (e.g., with relation strategies). Future work may explore integrating ontologies in representing nested relationships and hypergraphs in our proposed framework. Such an integration will facilitate scalable and semantically rich knowledge graphs that support further discovery in Alzheimer’s Disease and other biomedical fields.

### Limitations of the study

As a case study, our analysis of limitations in pairwise relationships is based on 56 AD articles (“Corpus” section). However, there are potential biases in these articles which may affect our conclusions. One potential bias is the selection criteria for the corpus. These 56 articles are in the AD field with a focus on amyloid-beta and selection criteria (see Details in “Corpus” section). However, articles with different focuses or with other selection criteria may be suitable for representation with other frameworks. Another potential bias is that these articles all come from the Year 2021. It is possible that AD-related articles before 2021 work perfectly with pairwise relationships only as knowledge representation. Whether there is a change in writing structures in AD articles or in experimental methodologies in AD research in different years is unknown.

However, since observations in 56 AD articles in this study are mostly biological processes, which are similar to other biomedical fields, we make an argument in the scope of biological knowledge representation, rather than AD only. We encourage researchers to examine the generalizability of representation frameworks in broader and more diverse datasets, such as stroke-related datasets. Such investigations will help refine and verify the applicability of general representation mechanisms like nested relationships and hypergraphs.

## Conclusion

In this study, we argue that employing simple graphs capturing pairwise relationships alone for biological knowledge representation has important limitations, particularly for the downstream task of inferring meaningful knowledge in literature-based discovery (LBD). Our systematic analysis of a recent binary LBD system in the context of Alzheimer’s Disease showed 7 types of limitations in standard knowledge graphs and 3 types of negative impacts on knowledge inference. Yet, pairwise relationships are found to be a foundational component for representation frameworks. More expressive knowledge representation strategies such as hypergraphs and nested relationships can make up for losses of pairwise relationships in biological knowledge representation. By integrating more semantically rich knowledge representation together with pairwise relationships, an LBD system can capture collective interactions and allow for nested entities. Spurious hypotheses can then be avoided and refined explanations for hypotheses can be generated. With biologically meaningful hypotheses and more granular explanations, significant resources to conduct biomedical experiments could be saved through better-justified predicted relationships. Our analysis should encourage LBD researchers to adopt more sophisticated knowledge representation strategies, ultimately helping domain experts to form biologically meaningful hypotheses and explanations for disease diagnosis, treatment, and mechanisms.

## Supplementary Information

Supplementary Material 1: Supplementary materials are provided in Figure 1, Figure 2, and Figure 3.

## Data Availability

Corpus and annotation are available at https://github.com/READ-BioMed/readbiomed-semantics/tree/main.

## Abbreviations

LBD: Literature-based Discovery
KG: Knowledge graph
AD: Alzheimer’s Disease
A$$\beta$$: Amyloid beta
CN: Common Neighbors
BEL: Biological Expression Language

## Footnotes

1 https://pubmed.ncbi.nlm.nih.gov/?term=Alzheimer%27s+Disease

2 Technically, undirected predicates are also possible. However, in practice these predicates are assumed to be directed in most extraction schemas and tools.

3 The group is part of the Florey Institute (www.florey.edu.au) and is led by Professor Colin Masters FRCPath, FRCPA, FFSc, FAA, FTSE, FAHMS, a distinguished neuropathology researcher.

4 https://app.diagrams.net/

5 https://github.com/READ-BioMed/readbiomed-semantics/tree/main

6 https://www.ebi.ac.uk/chebi/

7 https://geneontology.org/
