# Supplementary material for: Enriched knowledge representation in biological fields: a case study of literature-based discovery in Alzheimer’s disease
Source: J Biomed Semantics. 2025 Mar 20;16:3. doi: 10.1186/s13326-025-00328-3 (PMC11924609; doi:10.1186/s13326-025-00328-3)
Supplement: Supplementary file 1 — Supplementary Material 1: Supplementary materials are provided in Figure 1, Figure 2, and Figure 3. [file 13326_2025_328_MOESM1_ESM.pdf]

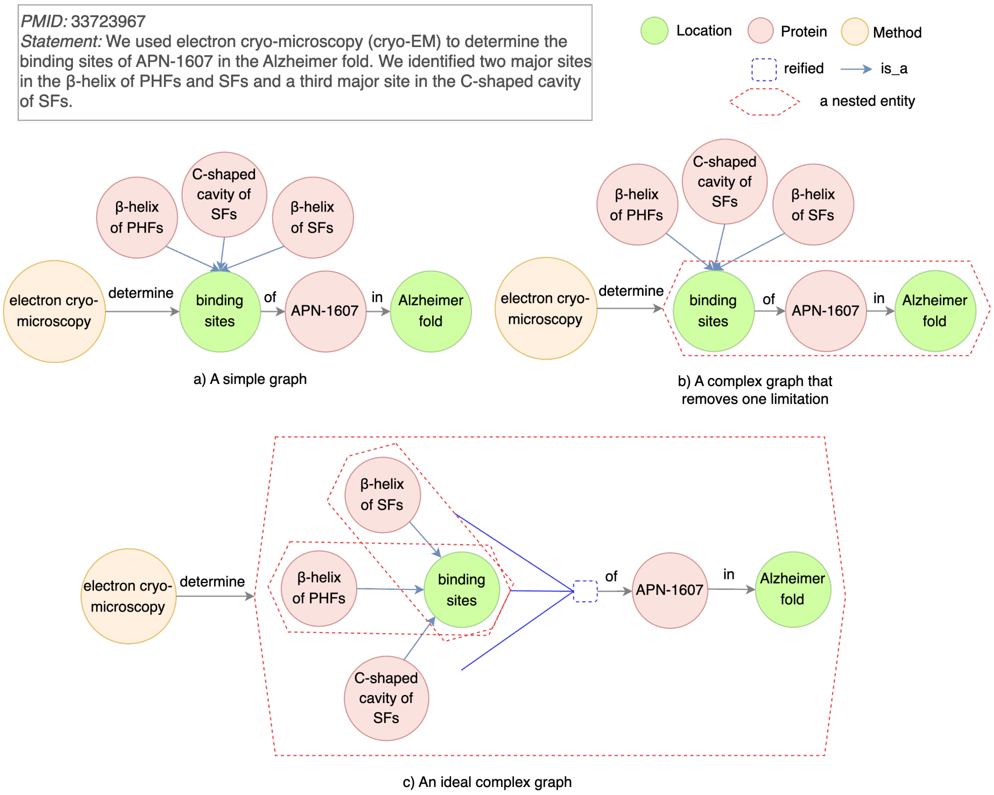

Fig. 1 A complete illustration of the pairwise relationship limitation type "Lack of context for a general concept". Top left (a): Each of three major sites (β`-helix of PHFs`, β`-helix of SFs`, and `C-shaped cavity of SFs`) is connected to a general concept `binding sites` with an `is_a` relation. `binding sites` is an object determined by a tool `electron cryo-microscopy`. Top right (b): One limitation of "Use of a method" (Details in Section ??) is removed with nested relationships. A nested entity of `binding sites of APN-1607 in the Alzheimer fold` is used as an object determined by a tool `electron cryo-microscopy`. Bottom (c): An ideal complex graph that further removes a limitation of "Lack of context for a general concept". A general concept `binding sites` and its contexts for three major sites are reified as an entity.

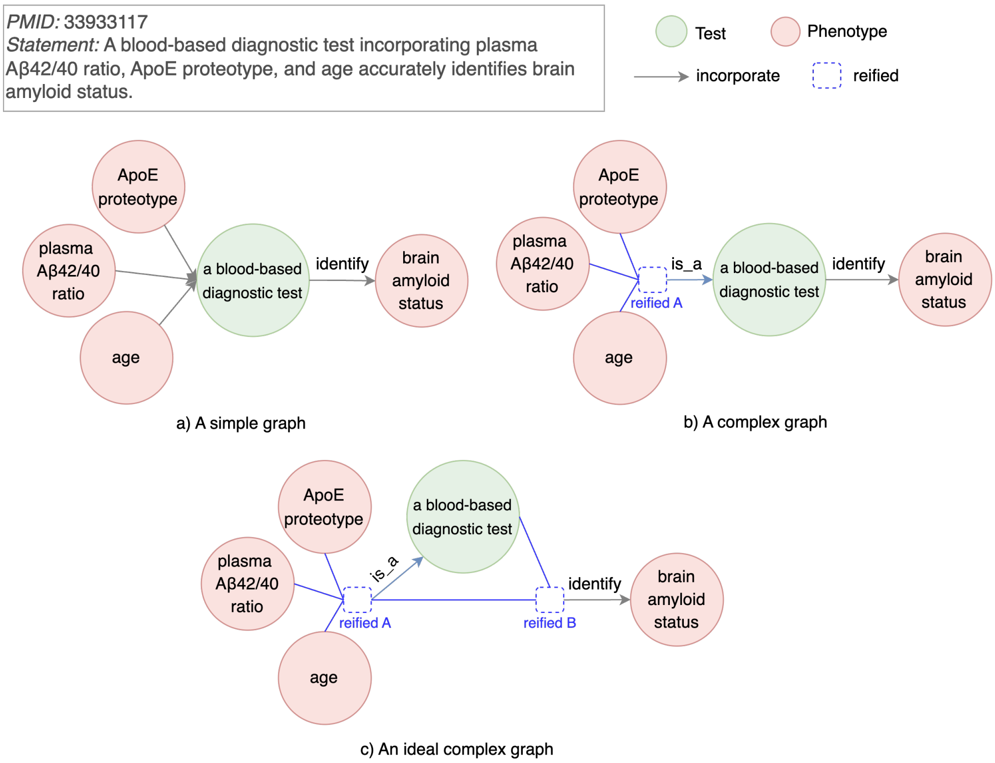

Fig. 2 A complete illustration of a paiwise relationship limitation type "More than two entities interacting together". Top left (a): Each of three biomarkers (`plasma A`β`42/40 ratio`, `ApoE proteotype`, and `age`) is connected to a general concept `a blood-based diagnostic test` with a relation of `incorporate`. `a blood-based diagnostic test` is a subject that identifies `brain amyloid status`. Top right (b): One limitation of "More than two entities interacting together" is removed with a hypergraph. Three biomarkers are reified as an entity (denoted as `reified A`). A reified biomarker is linked with a general concept `a blood-based diagnostic test` with a relation of `is_a`. Bottom (c): An ideal complex graph that further removes a limitation of "Lack of context for a general concept" (Details in Section **??**). `reified A` that consists of three biomarkers, as well as a general concept `a blood-based diagnostic test` are reified as another entity (denoted as `reified B`). `reified B` is used as a subject that identifies `brain amyloid status`.

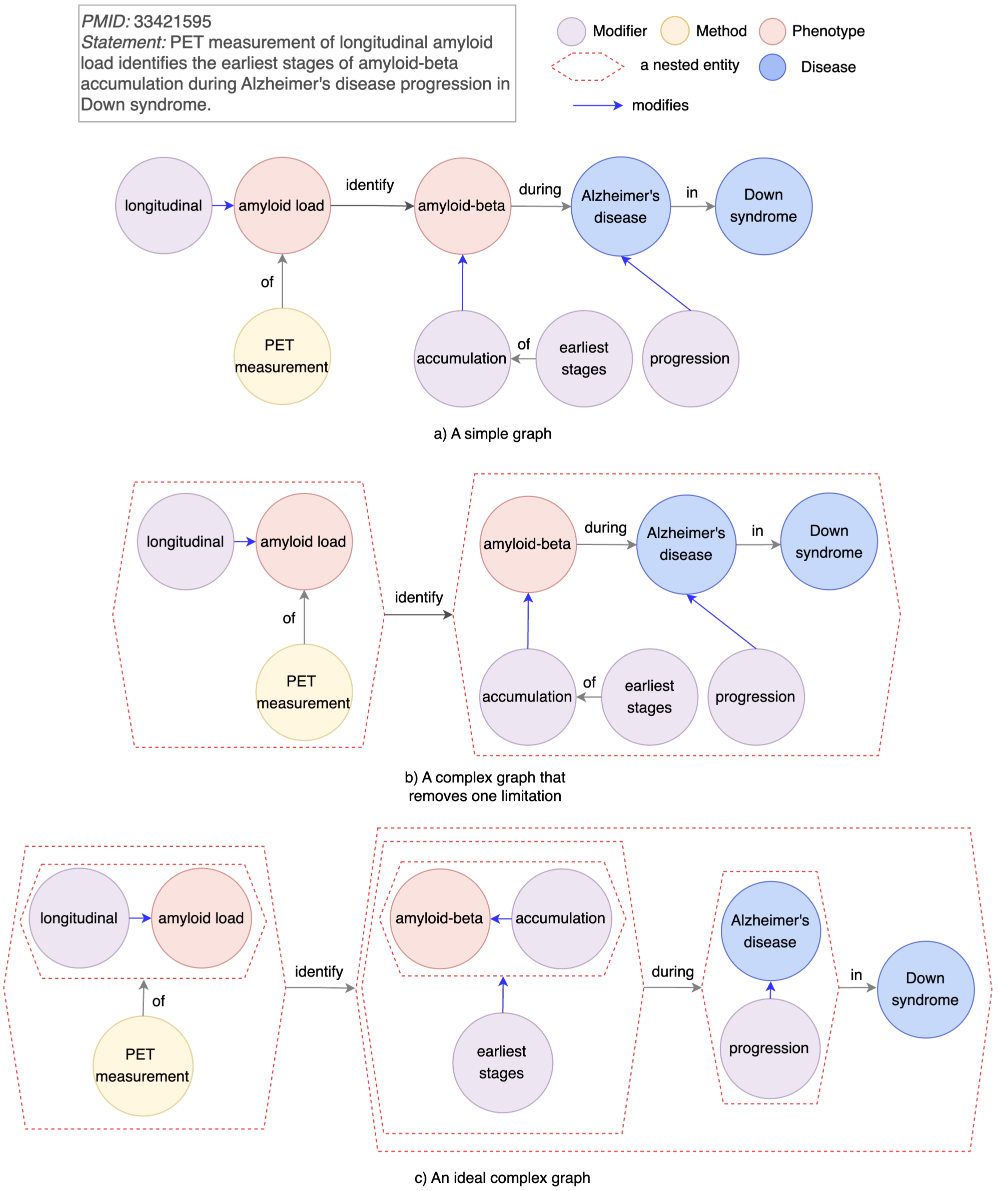

Fig. 3 A complete illustration of the pairwise relationship limitation type "Mechanism/process from a modified entity"". Top (a): A set of pairwise relationships between modifiers (`longitudinal`, `accumulation`, `progression`, and `earliest stages`) and corresponding entities (`amyloid load`, `amyloid-beta`, `Alzheimer's disease`, and `earliest stages of amyloid-beta accumulation`). `amyloid load` is used as a subject to identify an object `amyloid-beta`. Middle (b): One limitation of "A specific context as a constraint" (Details in Section **??**) is removed with nested relationships. `PET measurement of longitudinal amyloid load` is grouped as a subject to identify a grouped object `the earliest stages of amyloid-beta accumulation during Alzheimer's disease progression in Down syndrome`. Bottom (c): Modifiers and corresponding entities grouped together as processes (`longitudinal amyloid load`, `amyloid-beta accumulation`, `Alzheimer's disease progression`, and `earliest stages of amyloid-beta accumulation`).
